# Hazard assessment of oil spills along the main shipping lane in the Red Sea

**DOI:** 10.1038/s41598-021-96572-5

**Published:** 2021-08-23

**Authors:** H. V. R. Mittal, Sabique Langodan, Peng Zhan, Shihan Li, Omar Knio, Ibrahim Hoteit

**Affiliations:** 1 Computer, Electrical and Mathematical Sciences and Engineering Division, King Abdullah University of Science and Technology, (KAUST), Thuwal, 23955-6900 Saudi Arabia; 2 Physical Science and Engineering Division, King Abdullah University of Science and Technology, (KAUST), Thuwal, 23955-6900 Saudi Arabia; 3 Department of Engineering, Faculty of Agriculture, Dalhousie University, Halifax, NS B3H 4R2 Canada

**Keywords:** Natural hazards, Ocean sciences

## Abstract

This study investigates the risk from oil spills along the main shipping lane in the Red Sea based upon oil spill model trajectories forced by the outputs of validated high resolution regional met-ocean data. Following the intra-annual variations in the met-ocean conditions, the results are presented by classifying the basin into three regions: northern, central and southern Red Sea. The maximum distance traveled by the slick is presented for 1, 2, 5, 10, 14 and 20 days after the commencement of a spill. Different measures of hazard assessment in terms of the concentration of beached oil alongside the corresponding probability maps are also analyzed. The volume fractions of beached, dispersed and evaporated oil, 20 days after the commencement of a spill are quantified. The Red Sea general circulation is characterized by rich mesoscale eddies, which appear to be the most prevailing dynamics in oil transport in the basin. Two case events are analyzed to closely examine the effects of the mesoscale circulations on the fate of spilled oil. The results of this study provide a comprehensive assessment of oil spill hazards in the Red Sea, stemming its main shipping lane and identifies the areas at high risk that require timely mitigation strategies.

## Introduction

**Figure 1:**
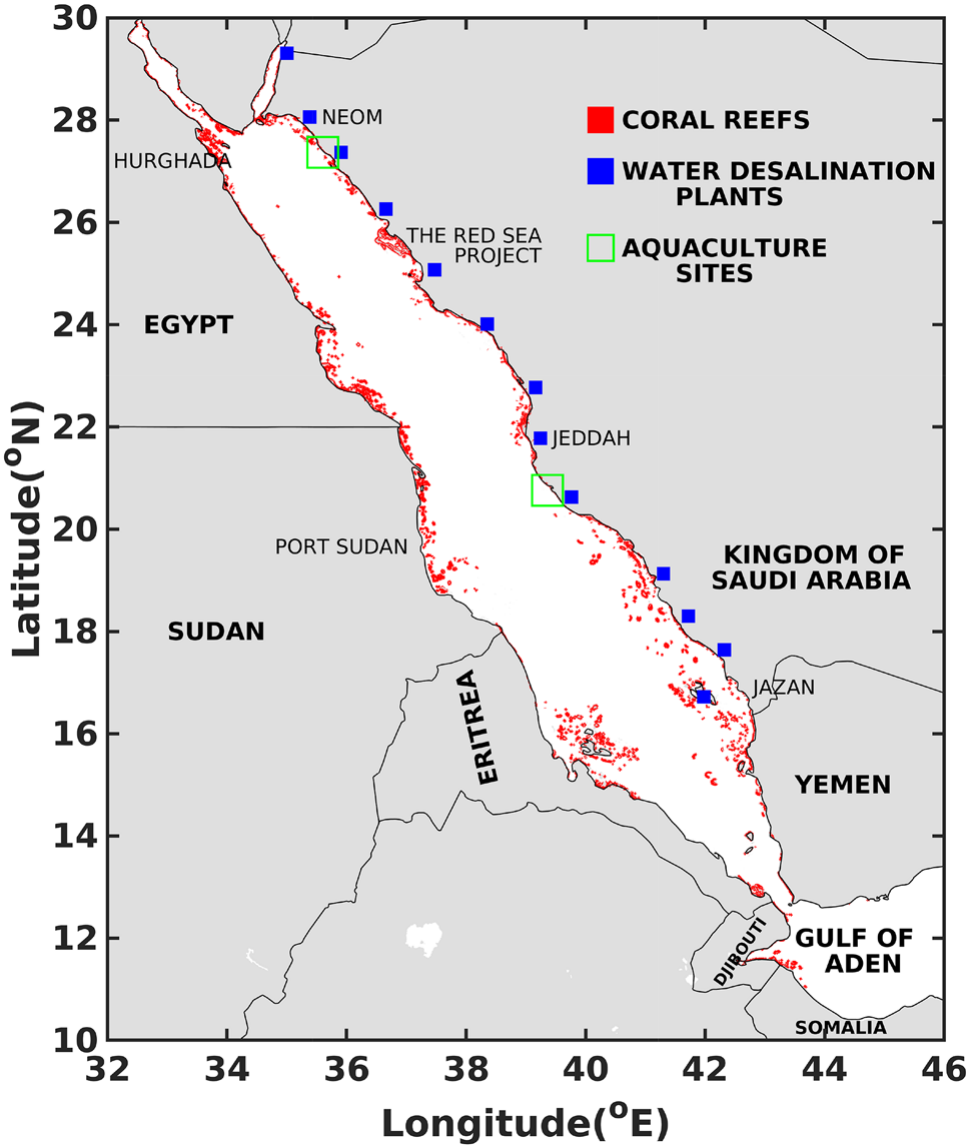
General map of the Red Sea illustrating the locations of coral reefs, largest cities, developments and installations along the coastlines. The figure was created on Matlab-r2020b (https://matlab-r2020b.software.informer.com). Coral reefs data are from open database Ocean Data Viewer (https://data.unep-wcmc.org/datasets/1).

The Red Sea is a long basin stretching 2250 km from the northwest to the southeast. Essentially closed at the north (except for the Suez Canal), the sea is open to the Gulf of Aden at the south through the narrow strait of the Bab-el-Mandeb and has an average depth of 490 m with a maximum depth of 2300 m. The Red Sea hosts a unique large marine ecosystem characterized by one of the second-longest and third-largest coral reef system in the world, thriving under one of the warmest and most saline conditions of the world oceans^1,2^. The Red Sea coasts are subjected to various developments, ranging from tourism to fishing, aquaculture, and are the main source of fresh water through desalination plants (see Fig. 1). The last decade has witnessed an unprecedented increase in the population and an acceleration in residential, commercial and industrial developments along the Red Sea coastlines e.g. NEOM and Red Sea Project^2^. Considered as one of the most heavily cruised waterways in the world, the Red Sea has been a main navigation route between East and West throughout the human history. Ancient Egyptian expeditions cruised the Red Sea basin to reach the Indian Ocean. Since the construction of the Suez Canal in 1869, the Red Sea has become one of the most important commercial pathways of the world, carrying maritime traffic between Europe and Asia.

Considering the massive transport of oil along its main axis, with a daily average of around one million barrels^3^, the Red Sea is consistently at risk of accidental oil spills. A particular incident led to an important oil pollution in February 2006 when at least 3300 tons of heavy fuel oil spilled into the northern Red Sea region. During another incident, 1100 tons of crude oil poured into the Suez canal at Bitter Lake in September 2006. In June 2010, an oil spill in the northern Red Sea polluted around 160 km of coastline, including tourist beach resorts in Egypt and the Jebel al-Zayt coast^4^. In October 2019, a vessel named Sabiti en route to Syria via the Suez Canal leaked oil while sailing over a stretch of about 150 km near the Saudi port of Jeddah^5^. Ship-borne transportation and the size of tankers are increasing by the day and this trend is likely to persist^6^. Therefore, a hazard assessment study of oil spills in the Red Sea is of paramount importance^7^.

Several hazard and risk assesment studies of oil spills have been conducted in many regions of the world, e.g. Alves et al.^8–10^ for the eastern Mediterranean, Liubartseva et al.^11^ for the southern Adriatic and northern Ionian (SANI) Sea regions, Olita et al.^12^ for the shorelines of a Mediterranean coastal archipelago, Shami et al.^13^ for four pilot areas located along the northern, eastern, and southern Mediterranean shorelines, Marty and Potter^14^ for the Canadian waters, Singh et al.^15^ for the Caribbean Sea, Canu et al.^16^ for Sicily coasts, and Kankara et al.^17^ for Chennai coasts in India. These studies generally assess the impact of oil spills from potential sources in the studied region such as oil and gas depots, offshore wells and operational ship lines. In the Red Sea, only a handful of studies have assessed hazards and risks from single oil spill accidents. Nasr and Smith^18^ performed oil spill simulations for four environmentally sensitive areas along the Egyptian coast in the Gulf of Suez using the S.L. ROSS oil spill model^19^. Ahmed et al.^20^ performed simulations using a two dimensional Eulerian model (^21^) to estimate the shortest time for oil to reach Bashayer Red Sea shoreline based on four spill scenarios, in summer and winter. Recently, Periáñez^22^ presented a Lagrangian model for the Red Sea and simulated oil trajectories from hypothetical release locations. The present study is the first to provide a comprehensive assessment of oil pollution for the whole Red Sea basin, namely from accidental spills stemming from the main shipping lane along its longitudinal axis.

The oil particles, before invading the shorelines, are prone to physical and chemical transformations referred to as weathering^23,24^. The times of migrations of oil particles, their dispersion in the water column and other weathering parameters strongly depend on the local climate conditions predominantly atmospheric winds, ocean currents and waves. The high mountain ranges on both sides of the Red Sea forces the wind to blow along its axis^25^. In summers, from April till October, a north west (NW) wind blows along the whole length of the sea, with speeds close to 10 $\hbox {ms}^{-1}$, and frequently exceeding 15 $\hbox {ms}^{-1}$^26^. In winters, the same northerly wind dominates over the northern part of the basin and south eastern (SE) winds associated to the north eastern (NE) monsoon in the Indian Ocean prevail over the southern Red Sea. The coexistence of northerly and southerly winds creates a convergence zone at the center of the Red Sea^27^. The larger and smaller valleys cutting across the bordering mountain ridges, lead to typical local winds relevant for characterizing the local wind regimes, e.g. wind passing through the Tokar Gap along Sudan coast in summer and westward blowing jets along the northeastern coast of the Red Sea in winter^25,28^.

The wave variability in the Red Sea is naturally associated with the dominant regional wind regimes^25^. Despite the moderate winds, a prolonged duration and the long fetch along the whole basin may generate waves as high as 3.5 m. During summer, the northwesterly winds prevailing over the whole Red Sea generate mean wave heights of 1–1.5 m in the north^25,29^, throughout the year. In winter, the monsoon associated winds generate mean wave heights of 2 m in the southern Red Sea and leads to a convergence zone in the central part when it meets the waves from the north^27^. The local wave regimes created by various transversal jets gradually merge with the dominant wave systems^26^.

The mean surface circulation in the southern Red Sea is largely modulated by the wind regime, which reverses between seasons. During winter, when the SE winds prevails, the surface inflow from the Gulf of Aden intensifies as a western boundary current in the southern basin and switches into an eastern boundary current north of 24$^\circ$ N^30^. In summer, the dominant NW winds push the surface outflow from the Red Sea into the Gulf of Aden^31^. In the central and northern Red Sea regions, the circulation is primarily characterised by multiple mesoscale eddies that tend to become more energetic during winter months following the development of intense baroclinic instabilities^32–34^, except for some strong semi-permanent wind-driven gyres that occur in summer^35,36^.

This study investigates the risk from oil spills along the main shipping lane in the whole Red Sea which are also analyzed in relation to the environmental conditions. The fate of oil is examined using the Modelo Hidrodin${\hat{a}}$mico (MOHID) oil spill model, a sub-model of the MOHID Water Modeling System^37^. MOHID is an open source, three dimensional water modeling system, developed by Marine and Environmental technology research center (MARETEC) at the Technical University of Lisbon^38–40^. The model is designed to simulate oil trajectories while taking into account weathering phenomena including evaporation, dispersion, sedimentation, dissolution, and emulsification. The MOHID oil spill model is driven by the outputs of validated high resolution met-ocean fields that are specifically generated for the Red Sea^2^. Since the surface weather and circulation conditions in the northern, central, and southern Red Sea regions exhibit distinct patterns over the seasons, the results are analysed separately for each of the three regions:

1. The northern region, where the northwesterly winds prevail throughout the year, and the circulation is characterized by mesoscale eddies where a cyclonic eddy dominates the circulation during winters.
2. The central region, where the convergence of southerly and northerly winds form in the winter, and the circulation is dominated by a rich mesoscale eddy variability.
3. The southern region, where the wind-driven currents dominate and follow a prevailing direction similar to the seasonally varying winds.

Oil spill simulations are performed starting at the first day of each month for 20 days, over four consecutive years spanning from 2013 to 2016, in order to provide a comprehensive picture of the risk due to the met-ocean variability in the Red Sea. The results of this study will largely help close the information gap in oil spill hazard assessment in the Red Sea. More specifically, the maps will provide new information about the coastal areas that are at risk of oil beaching, most vulnerable months and the minimum time for the oil to reach a particular location. This will support the authorities in marking areas at risk and accordingly design pre-deployment of booms, boats equipped with skimmer machines, vacuum trucks and dispersals in high-risk areas to setup the first responses for mitigation and cleaning operations.

The study is organised as follows. Section "Data sets and methodology" describes the met-ocean datasets, the oil spill model and the setup of the simulations. Section "Oil spill hazard analysis" presents the hazard analysis for the different regions of the Red Sea. A summary of the main findings is presented in "Summary" section.

## Data sets and methodology

The details of the met-ocean fields (wind, waves and currents) and the oil spill model are presented in this section, which also describes the experimental setup, and outlines the methodology adopted for presenting the hazard maps.

### The met-ocean datasets

The oil spill simulations are carried out based on the outputs of a validated met-ocean reanalysis fields generated by specifically customized models for the Red Sea^2^. The met-ocean fields were demonstrated to well describe the the Red Sea general oceanic and atmospheric circulations at the highest available resolutions^2,27,28,41^. The zonal and meridional winds were extracted from the in-house 5 km regional atmospheric reanalysis generated over the period from 1980 to 2020 using the Weather Research Forecasting (WRF) model assimilating all available regional observations (see details in^28,41^). WRF initial and boundary conditions are obtained from European Centre for Medium-Range Weather Forecasts (ECMWF) reanalysis Interim (ERA-I^42^) data. The WRF downscaling simulations were performed using the consecutive daily re-initialization method over 36-h periods^28,41^. The first 12-h period was neglected as a spin up and the remaining 24-h data was combined to generate a long-term reanalysis for the Arabian Peninsula. The wave conditions in the Red Sea were reconstructed using the WAVEWATCH III (WWIII) model forced with the aforementioned high-resolution WRF reanalysis winds^26^ on a regular grid of 1 km resolution.

The 3D ocean currents were simulated using the MIT general circulation model (MITgcm^43^) implemented at approximately 1 km grid resolution with 50 vertical layers. The model is forced with the aforementioned WRF reanalysis fields and the European Union Copernicus Marine Service Information (CMSEM) global ocean reanalysis fields^44^ at the open boundary in the Gulf of Aden on a 6-h and 24-h basis, respectively. The outputs of the Red Sea MITgcm have been extensively used to study the general and overturning circulations^30,31^, mesoscale eddies characteristics^33,34,36^, internal/baroclinic tides^45^, deep-water formation events^46^ as well as chlorophyll variability^47,48^.

The readers are referred to Hoteit et al.^2^ for a detailed description and validation of the WRF, WWIII, and MITgcm fields.

### The oil spill model

In MOHID, the oil fate and behavior is integrated with a Lagrangian module. The Lagrangian module simulates the spatio-temporal evolution of oil particles. The hydrodynamic module incorporates met-ocean data described in the previous section. The oil weathering sub-modules simulate the evolution of the oil intrinsic processes and its properties (density, viscosity, etc.). These properties along with many different processes such as oil spreading, evaporation, dispersion and emulsification, are accounted for in MOHID. Simulations of oil evaporation are described by the algorithms of Stever and Mackay^49^. Dispersion and emulsification processes are represented based on algorithms by Mackay et al.^50^. Oil dissolution and sedimentation are not considered to eliminate their effect on the oil mass balance.

The computational grid is Cartesian and uniformly spaced in the horizontal (lon-lat) and non-uniformly spaced vertical layers. The grid covers the entire Red Sea between longitudes $32^\circ$ and $46^\circ$ and latitudes $10^\circ$ and $30^\circ$ up to a depth of approximately 2746 m. The longitudinal and latitudinal axes are divided into 1401 and 2001 equally spaced nodes respectively, using a grid resolution of approximately 1 km in both axes. The met-ocean input fields for the model, which include the daily averaged 3D ocean currents, hourly winds, wave height and wave period are specified on the same horizontal and vertical grids. To properly describe the weathering processes, which vary over very short time scales, the time step of the oil module is set to 60 s. The time step of the Lagrangian module is set to 3600 s. To account for the impact of atmospheric winds on the spread and trajectories of the oil slick^51–53^, surface advection due to winds (wind drag effect) is also incorporated with a fixed value of $3\%$.

From each release location, MOHID outputs the temporal evolution of the global oil mass, volume and its properties such as density and viscosity, amount of oil evaporated and dispersed as well as the amount of oil beached and their beaching times. The outputs also encompass the location, mass, volume and density of each oil particle at desired time steps. The performance and accuracy of MOHID for oil spill modeling has already been demonstrated in many previous studies and ocean basins; see for example^54–57^.

### Setup of the oil spill simulations

**Figure 2:**
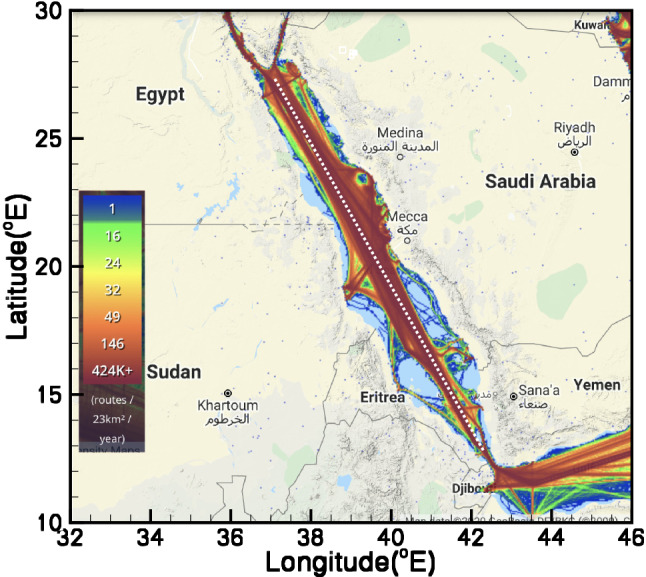
Contours depicting the marine traffic density and the release locations (shown in white dots along the longitudinal axis) in the Red Sea. The density is defined for the number as ships per 23 square kilometers averaged over a year (https://www.marinetraffic.com). The figure was created on Matlab-r2020b (https://matlab-r2020b.software.informer.com).

The density map of the vessels traveling across the Red Sea depicted in Fig. 2, suggests that the heaviest shipping traffic lines are recorded along the longitudinal axis of the Red Sea. Considering this massive transportation of oil across this axis, a uniform distribution of release locations, distanced approximately 20 km apart on the surface, on a approximately 1738 km long stretch of the longitudinal axis of the Red Sea is considered, depicted as white dots in Fig. 2. The 87 release locations ($\hbox {RL}_i:i=1:87$) selected for this study, lying between longitudes $42.6^{\circ }$ and $34.5^{\circ }$ and latitudes $14^{\circ }$ and $27.4^{\circ }$, are divided into the following three sets. Locations numbered $\hbox {RL}_1$–$\hbox {RL}_{{26}}$ lie in the southern region, $\hbox {RL}_{{26}}$–$\hbox {RL}_{{53}}$ in the central region and $\hbox {RL}_{{53}}$–$\hbox {RL}_{{87}}$ in the northern Red Sea. Instantaneous spills from each release location starting the first day of each month are conducted for four consecutive years spanning from 2013 to 2016. The Arabian crude oil with a specific gravity of $26^\circ$ API is chosen as the hypothetical released oil. This is a medium oil with relatively low viscosity and oil-water interfacial tension (before weathering). Therefore, the surface slick of this oil can be dispersed into the water column within a relatively short time. 2500 $\hbox {m}^3$ of oil by means of 500 particles are released from each location. The position of the oil slick is presented in terms of superimposed maps for 1, 2, 5, 10, 14 and 20 days after the commencement of the spill for all three regions separately. These maps represent a cumulative distribution of oil slicks in terms of the maximum extent that might be reached by the oil. Further, the positions of only those oil particles that are beached at the shorelines, at the end of each simulation, are plotted for each month. Different measures of hazard assessment in terms of the concentration of beached oil alongside the corresponding probability maps are also presented. At the final step, fractions of dispersed and evaporated oil, and the volume fraction of oil invading the shorelines, 20 days after the commencement of the spill are sketched in terms of box plots for all four investigated years. The box plots represent the median, which is the midpoint of the range of a set of values; the upper and lower quartiles, and their minimum and maximum values. These plots provide a visual map of the seasonal distributions of the above mentioned quantities for all four investigated years.

## Oil spill hazard analysis

**Figure 3:**
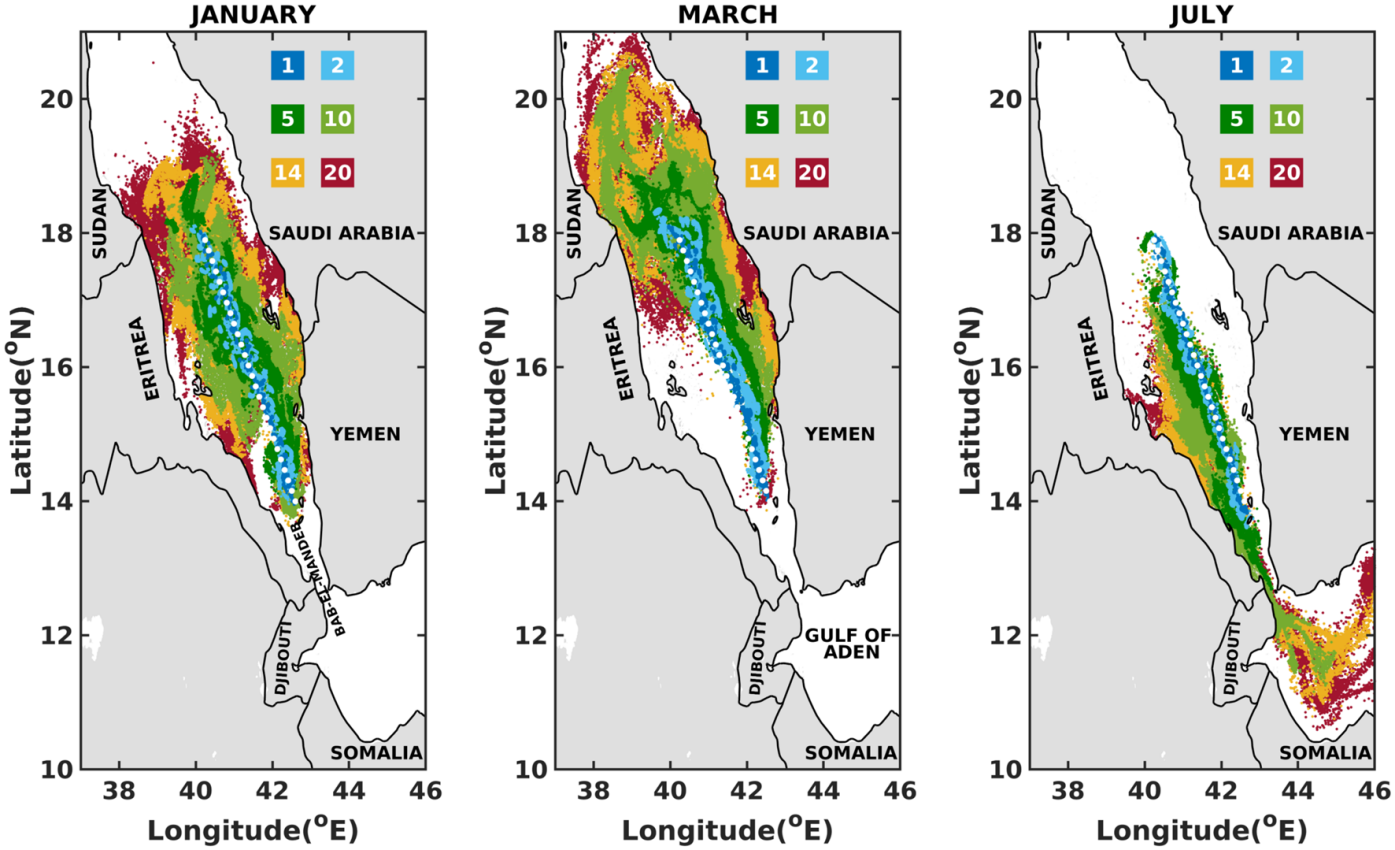
Superposition maps for the one (1), two (2), five (5), ten (10), fourteen (14) and twenty (20) days after the commencement of the spill, in the Southern Red Sea. The figures were created on Matlab-r2020b (https://matlab-r2020b.software.informer.com).

**Figure 4:**
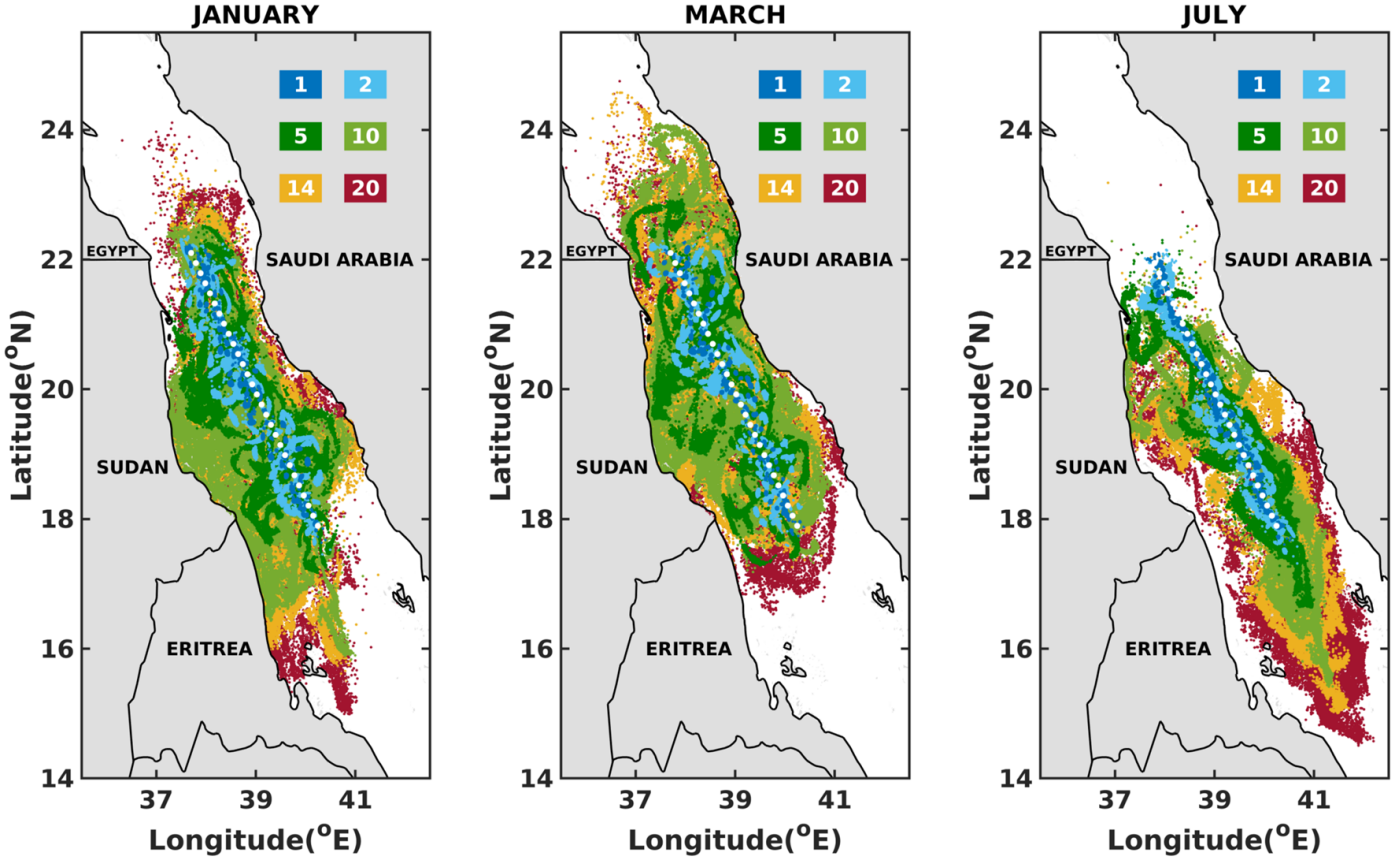
Superposition maps for the one (1), two (2), five (5), ten (10), fourteen (14) and twenty (20) days after the commencement of the spill, in the Central Red Sea. The figures were created on Matlab-r2020b (https://matlab-r2020b.software.informer.com).

**Figure 5:**
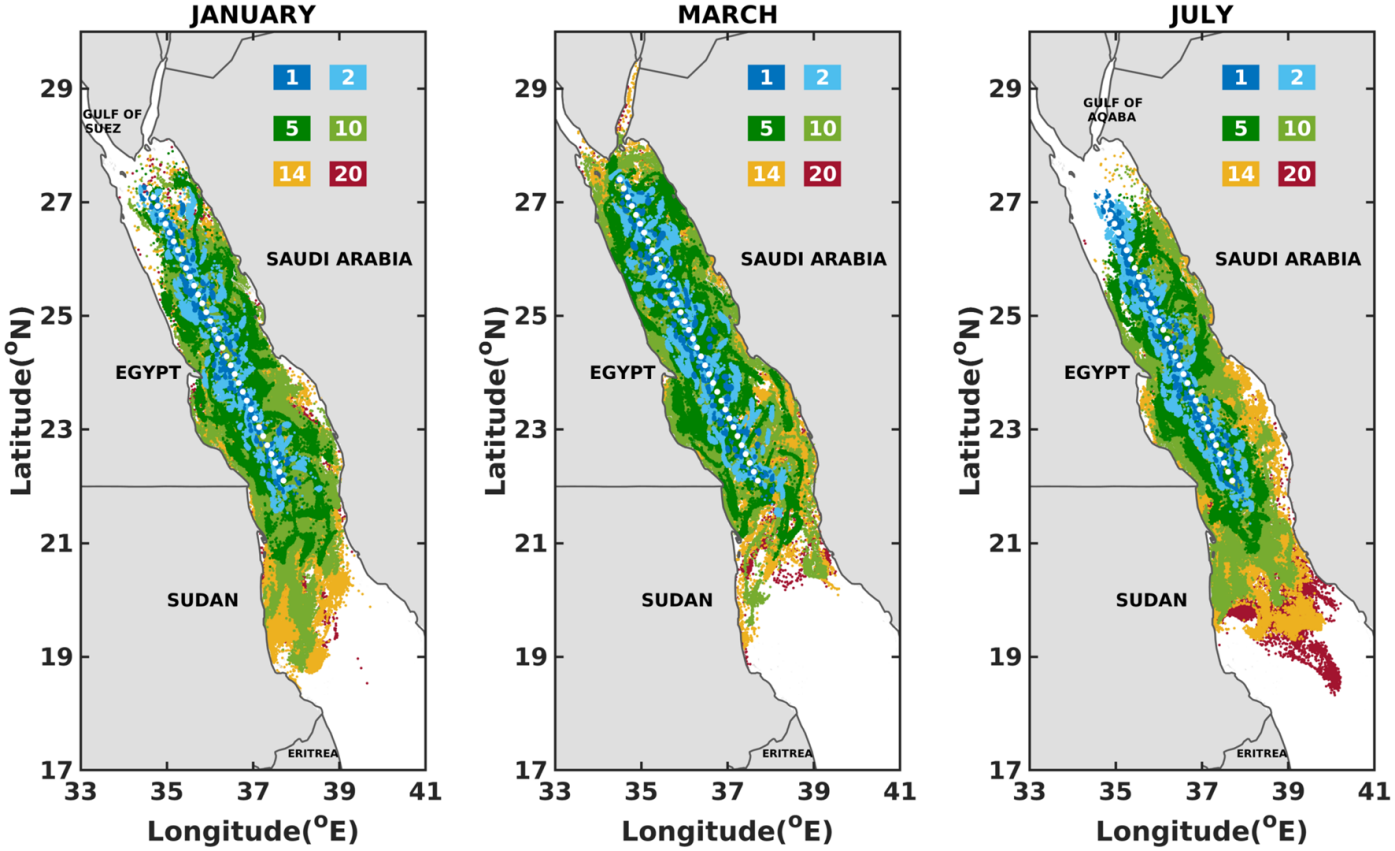
Superposition maps for the one (1), two (2), five (5), ten (10), fourteen (14) and twenty (20) days after the commencement of the spill, in the Northern Red Sea. The figures were created on Matlab-r2020b (https://matlab-r2020b.software.informer.com).

Figures 3, 4, and 5 and supplementary Figs. S1–S3 sketch the superimposed maps of oil slicks for 1, 2, 5, 10, 14 and 20 days after the initiation of the spills. A clear pattern from these figures is the rapid drift of oil particles in distinct directions as early as 2 days after the commencement of the spill. This suggest a strong and a fast impact of the atmospheric and oceanographic conditions on the spread and drift of oil in the Red Sea.

An important observation from the superimposed maps in the southern Red Sea region presented in Fig. 3 and supplementary Fig. S1 is that the oil slicks tend to shift from west (Eritrea–Sudan coastlines) during summers (till August) to east (Yemen–Saudi Arabian coastlines) at the onset of winters (from September). This can be associated with the dominant atmospheric winds and surface currents, which reverse from northwesterly to southeasterly following the Indian monsoon^30^. The southern basin is shallow and narrow, where wind-driven currents dominate and follow a prevailing direction similar to the seasonally varying winds^30,31,34^. During summers (June–September), the spread and drift of the slicks to the Gulf of Aden is prominent due to the prevailing currents that tend to flow out of the Red Sea towards the Gulf of Aden^58,59^. Consequently, the Djibouti–Somalian coastlines are at higher risk of oil beaching during summers. The spread and drift of the slick to the Gulf of Aden stops at the onset of winters (since October), as a result of the prevailing northward currents from Bab-el-Mandeb strait^31^. In the whole southern region of the Red Sea, the most vulnerable coastlines are those of Eritrean and Yemen, which seem to be affected as early as 5 days after the onset of the spill for about 5 months a year. On the other hand, the Sudan and Saudi Arabian coastlines seem at lower risk of oil beaching for about 5 months in a year, i.e. (June–September, December) and (June–October) respectively. For the remaining months, the oil particles take around 14 days to reach the Sudan–Saudi Arabian coastlines.

The simulations for the central Red Sea, plotted in Fig. 4 and supplementary Fig. S2, show broadening of slicks in the east-west directions, beaching the coastlines of Eritrea, Sudan and Saudi Arabia. This is mainly driven by the convergence zone in winter generated by the opposing winds^27^ and the complex eddy-dominant circulation system throughout the year^32^. The cyclonic/anticyclonic flow promotes rapid migration of oil towards the coastlines on both sides of the Red Sea. In this region, currents are observed to flow in different directions over almost the whole year, featured with strong eddies near the Eritrea-Sudan border in summers. Drifting of slicks away from the Sudan/Erirtrea border is prominent during the month of July due to the high intensity eastwards winds passing through the Tokar gap at around $18^\circ \,\, \hbox {N}$^35,36^. Further, the northwesterly winds prevailing over the region in summer enhances the spread of the oil slicks towards the south. In the central Red Sea region, the coastlines of Sudan are at risk of oil beaching for around 11 months a year as early as 5 days after the commencement of a spill. On the other hand, the Saudi Arabian coasts are at risk of beaching by oil particles practically during all months. In this case, the minimal arrival time is around 5 days for 6 months in a year.

Throughout the year, in the northern Red Sea region, superimposed maps in Fig. 5 and supplementary Fig. S3 report oil slicks broadening towards both the western and eastern coasts of the Red Sea. The coastlines of Egypt, Sudan and Saudi Arabia are prone to beaching starting as early as 5 days after the commencement of the spill. This can be attributed to the chaotic circulations in the northern region featuring mesoscale eddies with varying directions as they meander, intensify and weaken. Furthermore, a northwards migration of slick towards the Gulf of Suez in the months of March and April is observed. In the northern Red Sea region, both the east-west coastlines of the Red Sea (Egypt–Sudan–Saudi Arabia) are prone to beaching during all months of the year with a minimum arrival time of 5 days.

**Figure 6:**
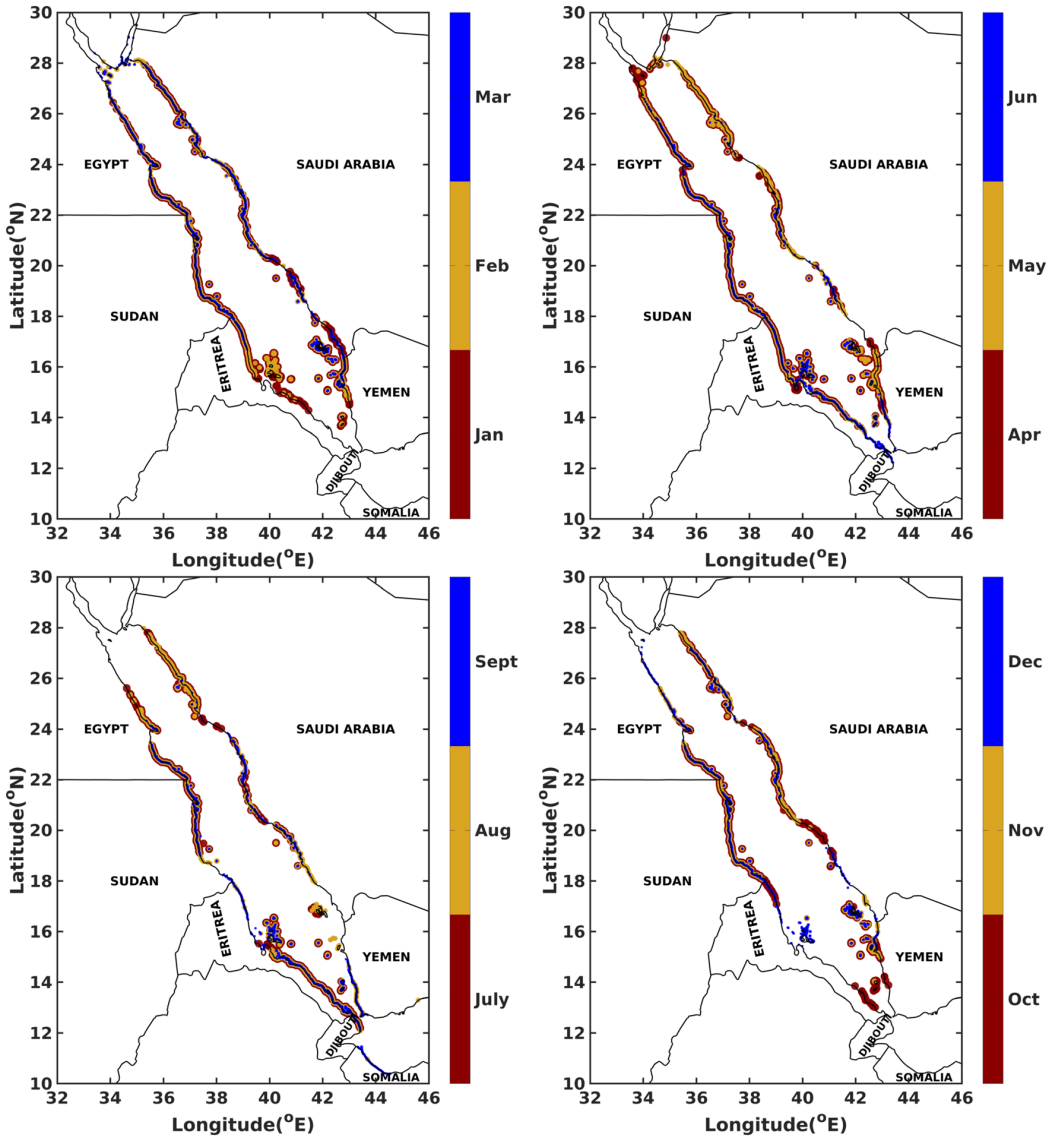
Superposition maps of the positions of oil particles beaching the shorelines in the whole Red Sea for all twelve (12) months, twenty (20) days after the commencement of the spill. The figures were created on Matlab-r2020b (https://matlab-r2020b.software.informer.com).

**Figure 7:**
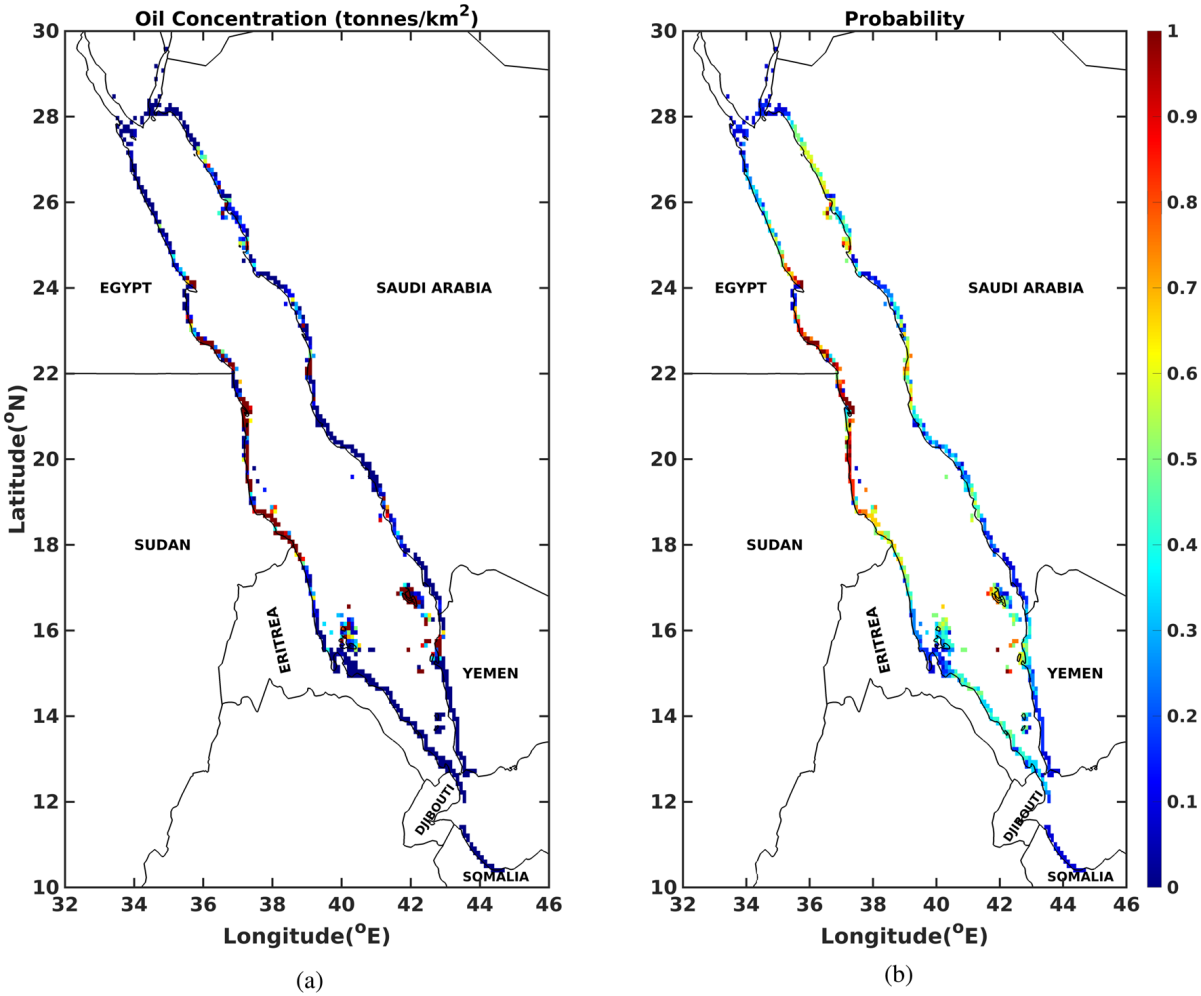
(**a**) Normalised cumulative concentrations of oil particles and (**b**) Probability map in terms of positions of oil particles, beaching the shorelines over all 12 months in the whole Red Sea, twenty (20) days after the commencement of the spill. The figures were created on Matlab-r2020b (https://matlab-r2020b.software.informer.com).

The hazard assessment in the Red Sea in terms of shorelines beached by oil, for each month, 20 days after the commencement of the spill is illustrated in Fig. 6. For almost all months a year, observations report that both east–west shorelines of the Red Sea seem at risk of oil beaching. From February to May, the shorelines along the Gulf of Aqaba and Suez are at risk of oil beaching. Djibouti and Somalian coastlines are at a risk of oil beaching only during the months of June–September and August-September, respectively. From April to September, the whole length of Eriterian coastline are at risk. During the months of July to September, the coastlines around Saudi–Yemen border (southern and northern coastlines of Saudi Arabia and Yemen respectively) are at low risk of beaching. Likewise, the coastlines of Yemen and Eritera seem to be at low risk of beaching during the months of July and November respectively. Figure 7a illustrates normalised cumulative concentrations of oil particles invading the shorelines, over the whole year, 20 days after the commencement of the spill. The southern coastlines of Egypt (adjoining the border of Sudan), almost the whole length of Sudanese coastlines and the beaches of the islands near the border of Saudi–Yemen, seem at risk of beaching with the highest oil concentrations. In contrast, the shorelines along Gulf of Aqaba/Suez and of Djibouti–Somalia receive the lowest oil concentrations. Information about the probability of oil particles beaching the shorelines over the whole year can be extracted from the corresponding probability map in Fig. 7b. It is observed that the probability of oil beaching is highest for the southern coastlines of Egypt (adjoining the border of Sudan), Sudan and beaches of the islands near the border of Saudi–Yemen, and lowest for the shorelines along Gulf of Aqaba/Suez and of Djibouti–Somalia.

**Figure 8:**
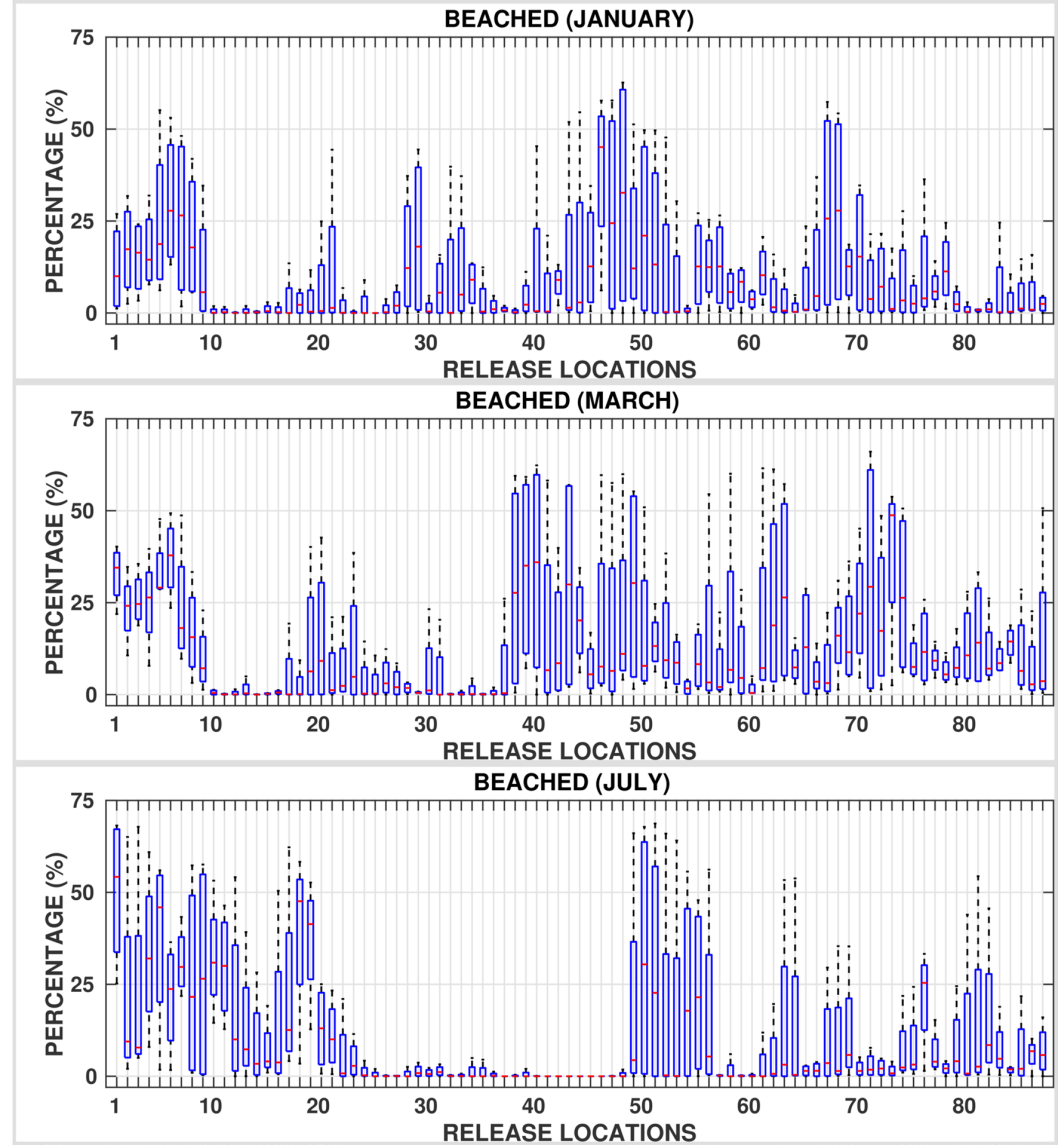
Percentages of oil beached, twenty (20) days after the commencement of the spill for each release location ($RL_i \;:\; i = 1:87$) for all the 4 years considered in simulations.

**Figure 9:**
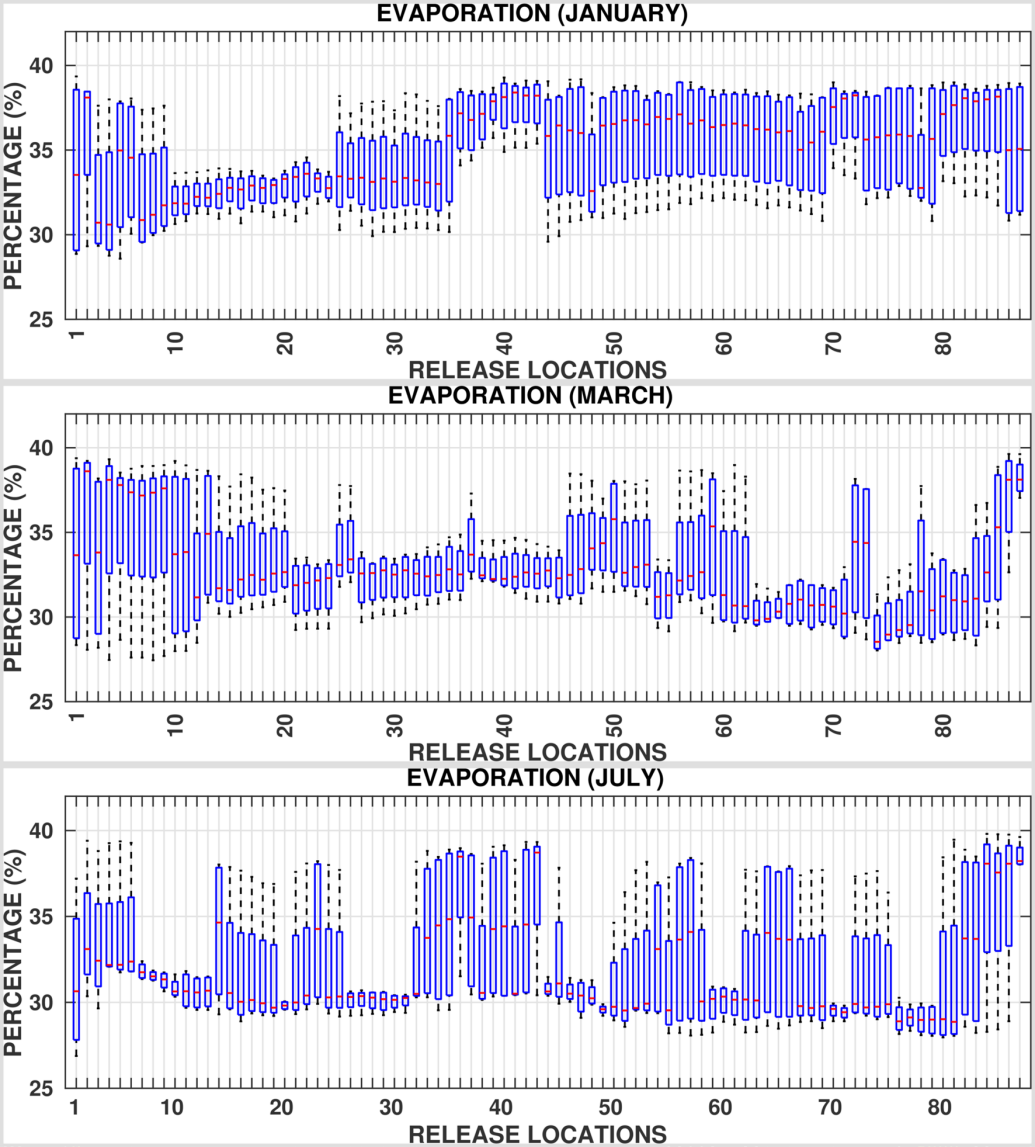
Percentages of oil evaporated, twenty (20) days after the commencement of the spill for each release location ($RL_i \;:\; i = 1:87$) for all the 4 years considered in simulations.

**Figure 10:**
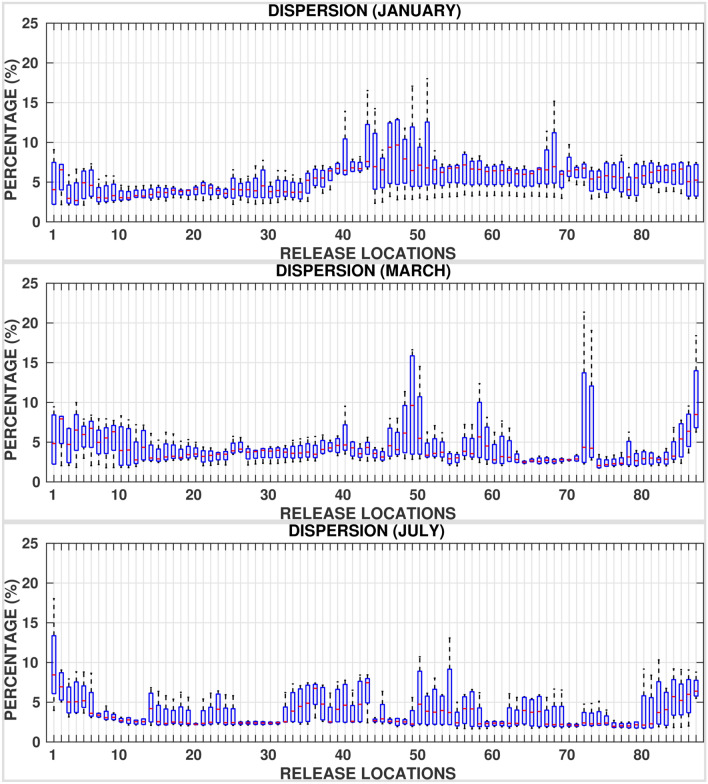
Percentages of oil dispersed, twenty (20) days after the commencement of the spill for each release location ($RL_i \;:\; i = 1:87$) for all the 4 years considered in simulations.

Figures 8, 9, and 10 and supplementary Figs. S4–S6 show the box plots of fractions of oil particles that beached the shorelines, evaporated and dispersed, respectively suggesting large variations. For instance, in the central Red Sea region, the volume fractions of beached oil vary from almost $0\%$ in one particular year to as high as $70\%$ in another year for a given location and month. This is due to the fact that the central region experiences the highest variability in the basin in terms of met-ocean conditions with opposing wind directions and active eddies. Further, eddies tend to trap a part of the spilled oil for longer periods, promoting longer persistence of oil on the surface of the ocean in the middle of the Red Sea near the spilled location. As such, the central region is at least risk of beaching by oil particles while experiencing the highest dispersion rates. The southern Red Sea region is at the highest risk of oil beaching amongst other regions, especially during the period between July and September. These trends are attributed to the close proximity of release locations to nearby shorelines, existence of numerous small islands, and the prevailing current directions. Relatively high mean evaporation, up to $40 \%$, is observed in winters over the whole Red Sea due to the high intensity of winds over the region. Further, the rates of dispersion are lower, less than $5\%$, in the southern and central Red Sea regions over roughly 10 months in a year.

### Spread of oil due to mesoscale circulation

Mesoscale eddies^33,34,58^ appear to be the most important dynamics governing oil transport in the Red Sea. To further assess the role of eddies in the transportation of spilled oil, two case events are examined during the year 2013. Oil concentrations are presented in the vicinity of a typical cyclonic eddy (CE) that dominates the circulation during winter in the northern Red sea region, and of a quasi-stationary anti-cyclonic eddy (AE) during summer in the central Red Sea region.

**Figure 11:**
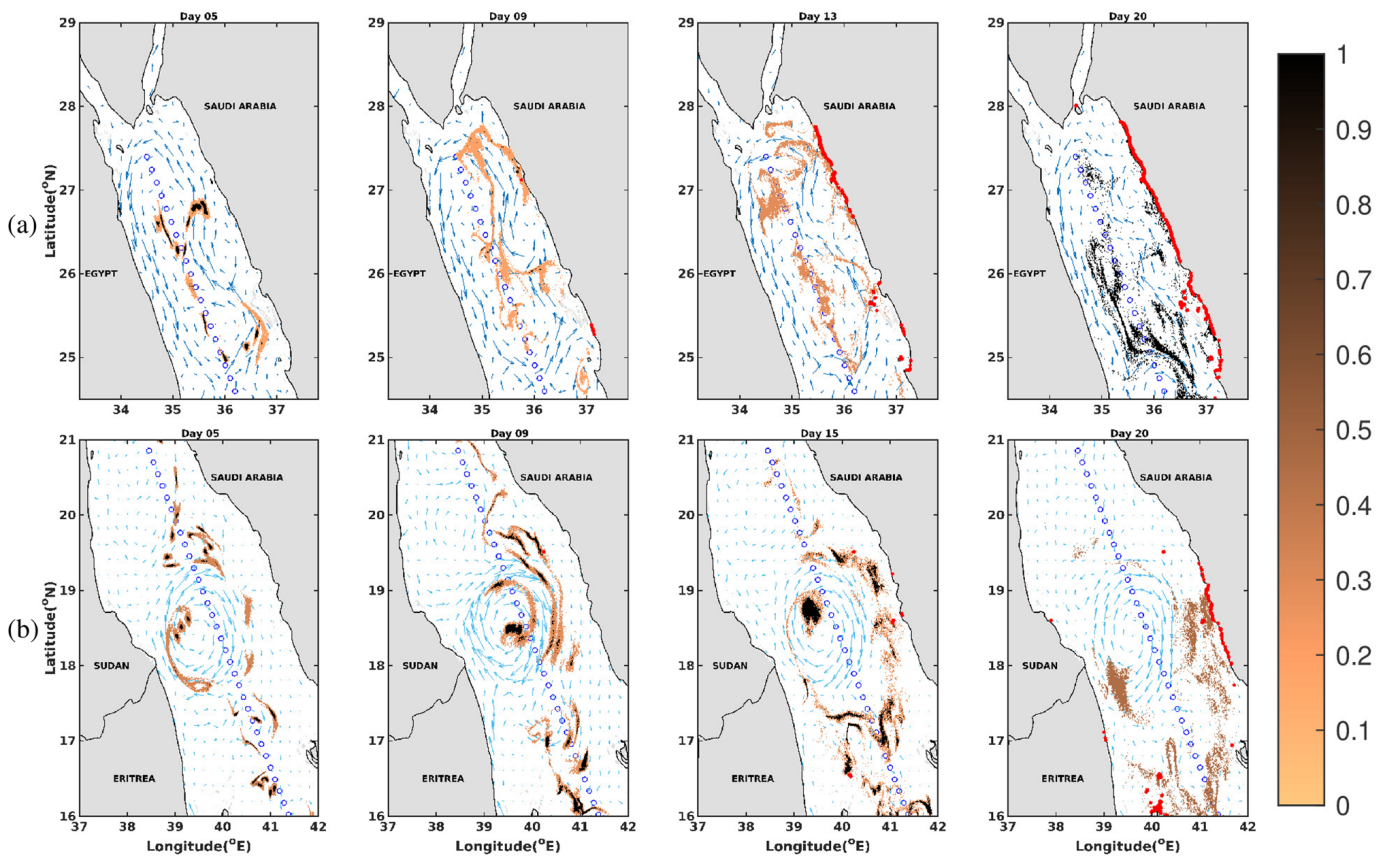
Snapshots of the plots of oil concentrations superimposing the surface currents vectors for the months of (**a**) November and (**b**) August, where the red markers indicate the beached oil particles. The figures were created on Matlab-r2020b (https://matlab-r2020b.software.informer.com).

In the first case on November 2013, the CE spanned the shorelines of the northern Red Sea between Egypt and Saudi Arabia, as illustrated by the surface currents in Fig. 11a. The oil concentrations after 5, 9, 15 and 20 days of the onset of the spill (Fig. 11a) suggest that the chaotic surface conditions resulting from the energetic circulations tend to consistently spread the spilled oil over a wide range, with some of the oil particles being entrained in the circulating eddy-like patterns, along the central axis of the Red Sea. Similar behavior was observed and reported in the Black Sea^60^. In particular, in the year 2013, these patterns tend to split the slick, transporting the oil particles towards the eastern coasts (Saudi Arabian coasts) of the Red Sea, within about 9 days after the onset of the spill. The second case involved an AE in the central Red Sea region on August 2013. The AE has a diameter of approximately 200 km and was generated by high intensity winds passing through the Tokar gap^35^, covered the basin between the shorelines of Sudan-Eritrea, and Saudi Arabia. As shown by the concentrations of spilled oil particles in Fig. 11b, the circulation tends to create a convergence zone where a large fraction of the spilled oil is trapped by the mesoscale features of the circulation, till Day 15. Relatively high oil concentrations are observed near the center of the AE between days 9 and 15, which reflects the accumulation of oil particles near the center of the eddy. Meanwhile, parts of the spilled oil develop into filaments and are transported towards the nearby coastlines in both east-west directions in the form of multiple slicks about 2 weeks after the onset of the spill.

A comparison between the snapshots at Day 20 in Fig. 11a, b suggests that the oil particles are transported in distinct directions (only east and both east-west respectively) in the Red Sea. This behavior can be attributed to the selected release locations with respect to the geographical locations of mesoscale circulations. A small displacement of the release location in the vicinity of an eddy can significantly alter the fate of oil slicks. Note that these experiments correspond to the simulations for one particular year (2013) and do not include the variations in the oil fate from 2014 to 2016. As discussed earlier in this study, the risk from oil spill should be assessed based on oil simulations from different years, which accommodates the interannual variability in the locations and strengths of the mesoscale circulations.

Eddies in the Red Sea generally grow with diameters similar to the width of the basin and give rise to strong currents in the upper layers^61^. A statistical analysis of the eddy variability^32^ suggests that these eddies tend to be mainly generated in the central and northern Red Sea regions, with stronger intensity during winter months when baroclinic instabilities are most pronounced^34^. Meanwhile, some eddies can be formed by wind stress, buoyancy forcing and boundary currents^36,61–63^, among which the most prominent event being the dipole generated by the Tokar wind jets during summer months in the central basin^35^. Compared to the general circulation in the Red Sea, eddies usually exhibit a larger magnitude in rotational velocity, which can lead to increased dispersal and mixing through a combination of advective stirring, stretching and contracting a coherent patch into typical filamental structures^33,34,59,64,65^. Therefore, mesoscale eddies play an important role in determining the fate of spilled oil in the Red Sea, with rapid spreading rate particularly in the central and northern Red Sea regions, as revealed by the results presented in Fig. 11.

## Summary

This study investigated the risks from accidental oil spills from ships cruising along the main shipping lane, along the longitudinal axis of the Red Sea. The fate of oil is predicted using the MOHID oil spill model driven by extensively validated high resolution regional met-ocean fields. Following the different weather conditions in the Red Sea, the results are presented by dividing the Red Sea into to northern, central and southern regions. Significant drift of oil particles, starting as early as 2 days after the onset of the spill, in distinct directions from all release locations is observed over the whole year. The central and northern basins are deeper, and their circulation is dominated by mesoscale eddies that are generally featured with larger velocities than the background flow. Consequently, the circulations in the central and northern Red Sea regions drive a rapid rate of transport and dispersal of materials across the coasts. As a result, oil slicks in the northern and central regions tend to migrate towards both the east-west coasts of the Red Sea beaching the shorelines of Egypt–Sudan–Saudi Arabia as early as 5 days after the onset of the spill for at least 11 months of a year. In contrast, the southern basin is shallower and narrower, where wind-driven currents dominate and follow a prevailing direction similar to the seasonally varying winds^30,31,34^. Consequently, the spilled oil are less likely to spread and exhibits significantly different fates according to the seasons. As a result, the coastlines of Eritrea–Yemen–Sudan–Saudi Arabia seem not prone to beaching for around 5 months in a year. The whole length of Sudanese coastlines including the adjoining southern coastlines of Egypt and the beaches of islands near the border of Saudi–Yemen are at the highest risk of oil beaching throughout the year. Moreover, these are at risk of invasion by the highest concentrations of oil.

The high seasonal variability in the met-ocean conditions in the Red Sea promotes large variations in the fractions of beached, evaporated and dispersed oil particles. The corresponding details are provided in terms of box plots corresponding to each release location for every month. These reveal that relatively high evaporation rates occur in the whole Red Sea region during winter. Furthermore, the highest beaching rates occur in the southern region over the whole year and a weaker dispersion occurs in the central and southern regions, for about 10 months of the year.

The presented hazard maps from oil spills along the main shipping lane provide valuable insights on the risk of oil pollution in the Red Sea. They contain comprehensive information about the direction of the spill and the coastal resources likely to be affected. The results of this study will assist the emergency teams in the design of efficient measures and strategies to mitigate the impacts of oil spills in the Red Sea.

Future work encompasses a long-term hazard assessment study to examine the interannual variability related to the met-ocean conditions, inclusion of other sources of risk (sea-ports, oil wells or pipelines), targeting specific accidents or regions (important developments/installations). Investigation of the measures that can lower the probability of oil particles beaching the shorelines by considering, for example, the variations in the positions of the release locations according to the weather conditions will also be studied.

## Supplementary Information

Supplementary Figures.
