# Supplementary material for: Hazard assessment of oil spills along the main shipping lane in the Red Sea
Source: Sci Rep. 2021 Aug 23;11:17078. doi: 10.1038/s41598-021-96572-5 (PMC8382762; doi:10.1038/s41598-021-96572-5)
Supplement: Supplementary file 1 — Supplementary Figures. [file 41598_2021_96572_MOESM1_ESM.pdf]

Supplementary Material

Risk assessment of oil spills along the main shipping lane in the Red Sea

H.V.R. Mittal[1], Sabique Langodan[2], Peng Zhan[2], Shihan Li[3], Omar Knio[1], and Ibrahim Hoteit*[2]

[1]Computer, Electrical and Mathematical Sciences and Engineering Division, King Abdullah University of Science and Technology, (KAUST), Thuwal 23955-6900, Saudi Arabia
[2]Physical Science and Engineering Division, King Abdullah University of Science and Technology, (KAUST), Thuwal 23955-6900, Saudi Arabia
[3]Department of Engineering, Faculty of Agriculture, Dalhousi University, Halifax, NS B3H 4R2, Canada

June 21, 2021

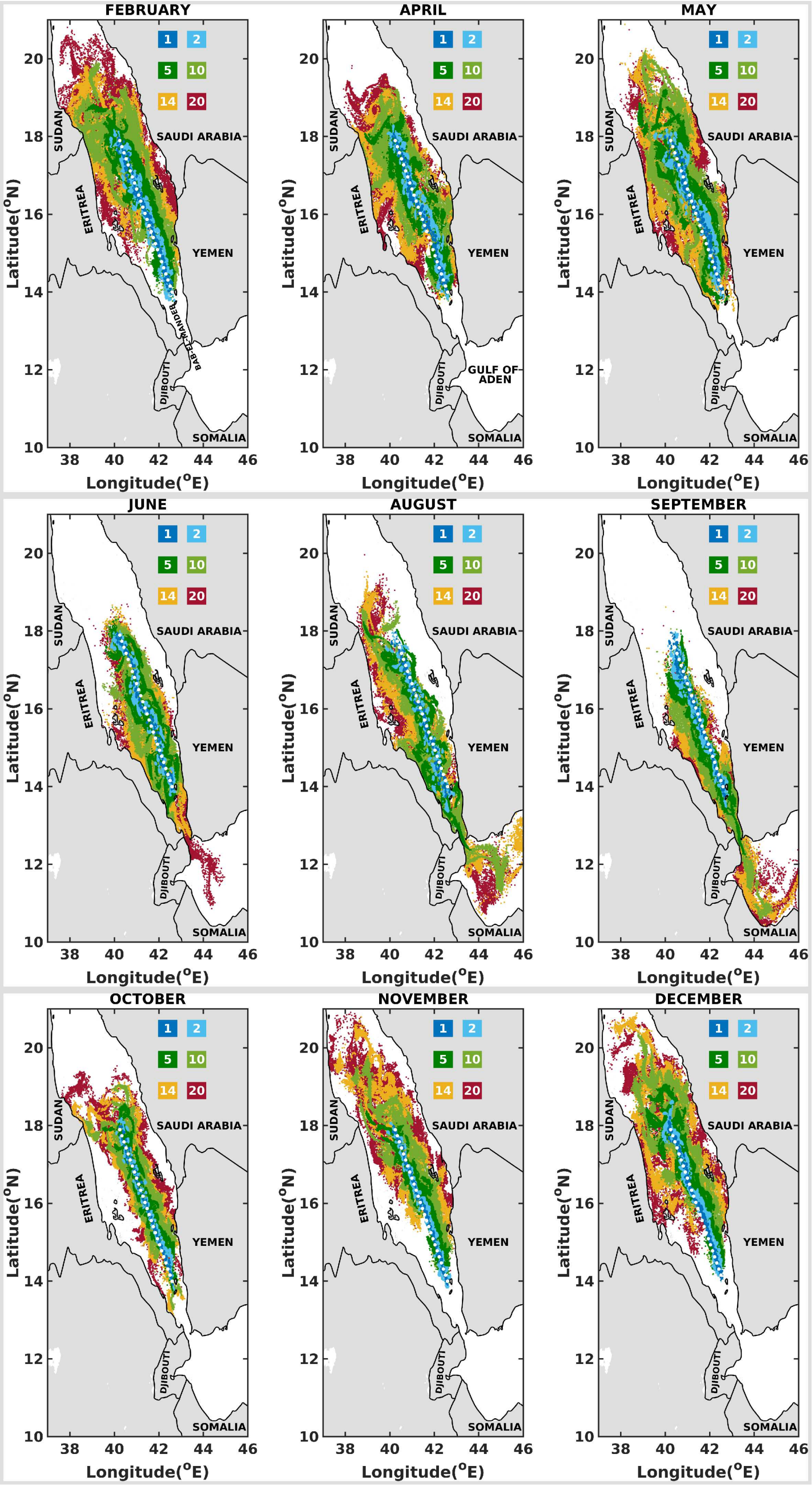

Supplementary Figure S1: Superposition maps for the one (1), two (2), five (5), ten (10), fourteen (14) and twenty (20) days after the commencement of the spill, in the Southern Red Sea. The figures were created on Matlab-r2020b (`https://matlab-r2020b.software.informer.com`).

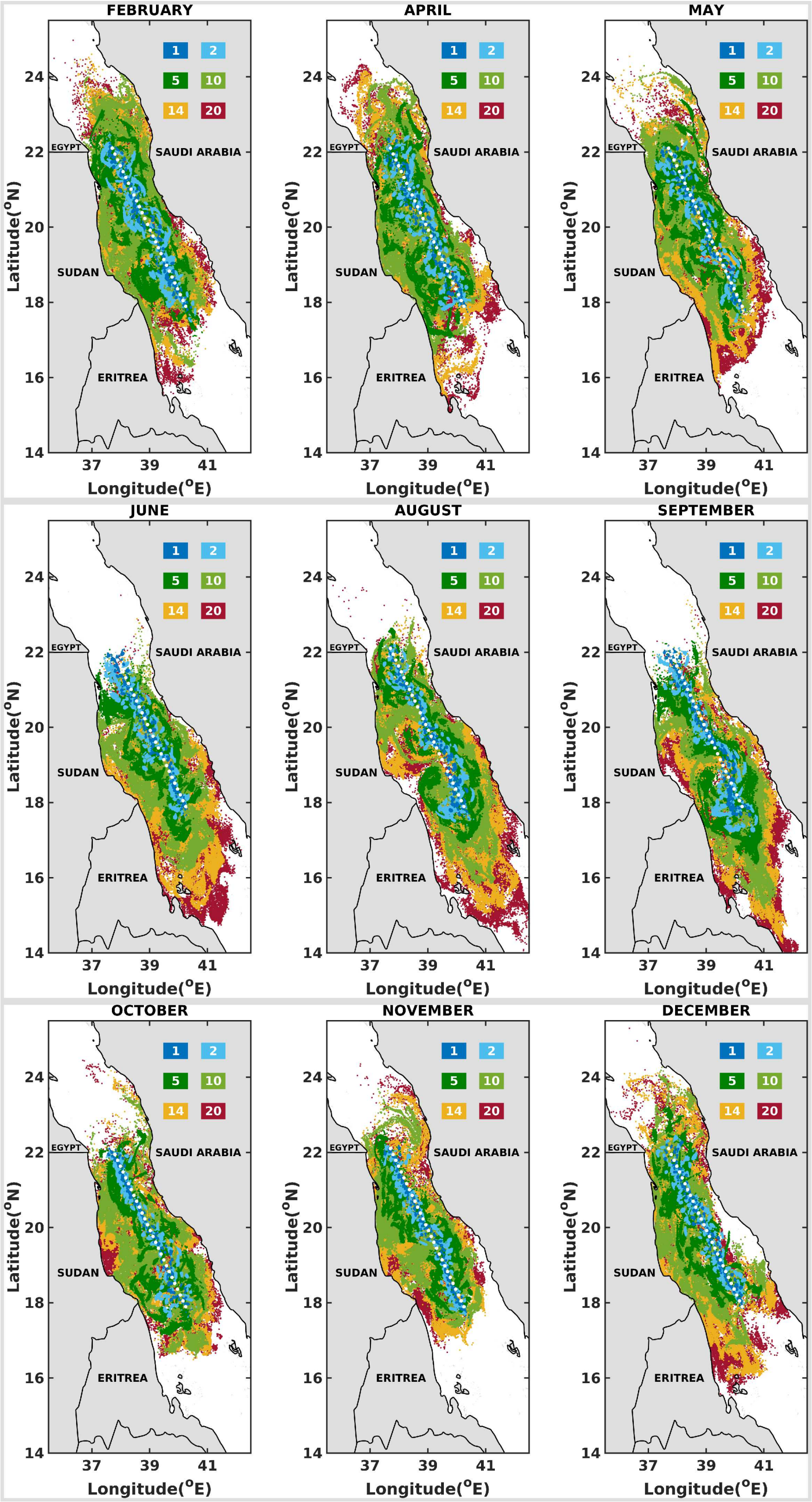

Supplementary Figure S2: Superposition maps for the one (1), two (2), five (5), ten (10), fourteen (14) and twenty (20) days after the commencement of the spill, in the Central Red Sea. The figures were created on Matlab-r2020b (`https://matlab-r2020b.software.informer.com`).

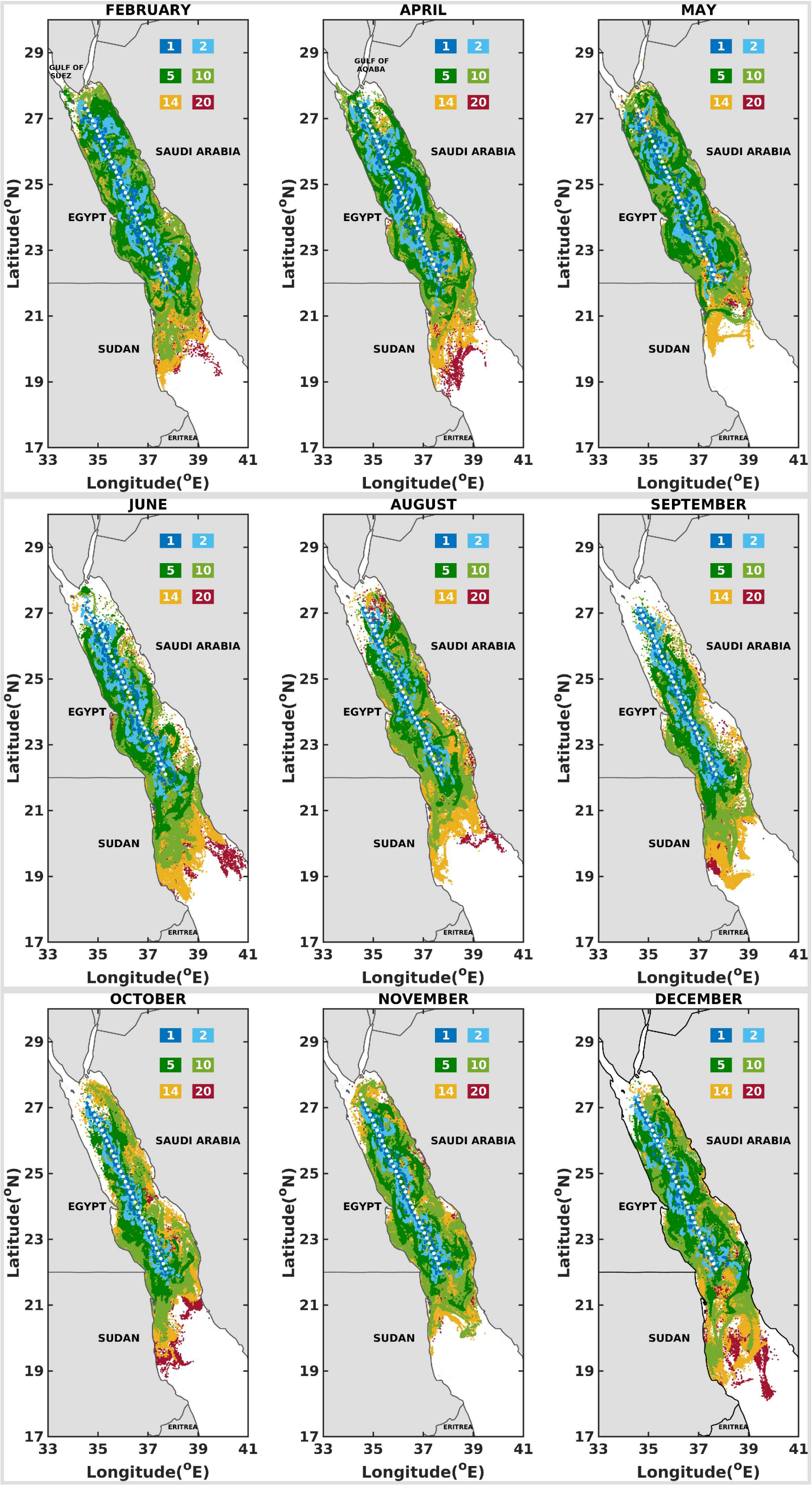

Supplementary Figure S3: Superposition maps for the one (1), two (2), five (5), ten (10), fourteen (14) and twenty (20) days after the commencement of the spill, in the Northern Red Sea. The figures were created on Matlab-r2020b (`https://matlab-r2020b.software.informer.com`).

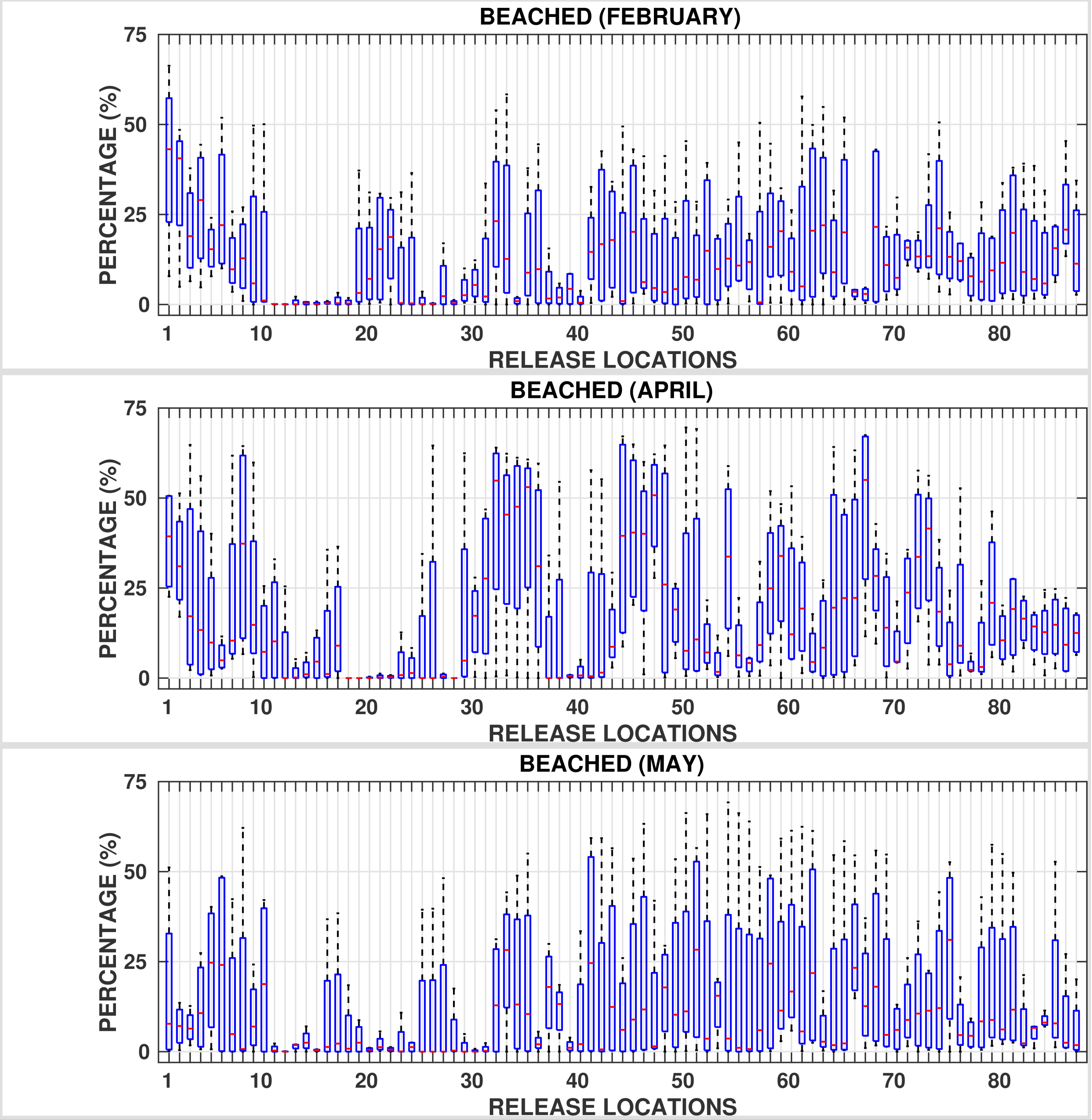

Supplementary Figure S4: Percentages of oil beached, twenty (20) days after the commencement of the spill for each release location ($RL_i \ : \ i = 1 : 87$) for all the four years considered in simulations.

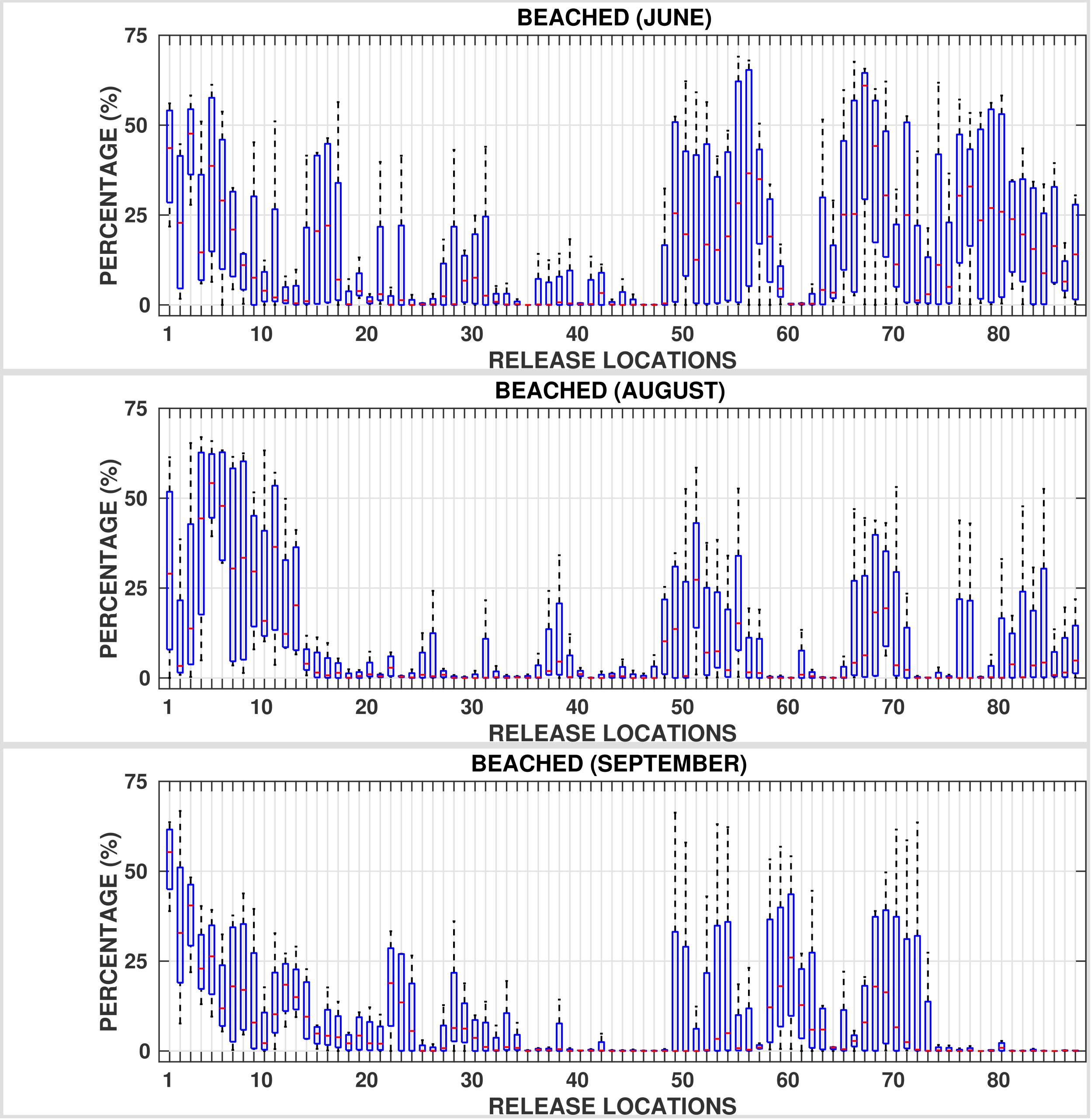

Supplementary Figure S4: Percentages of oil beached, twenty (20) days after the commencement of the spill for each release location ($RL_i\ :\ i = 1 : 87$) for all the four years considered in simulations.

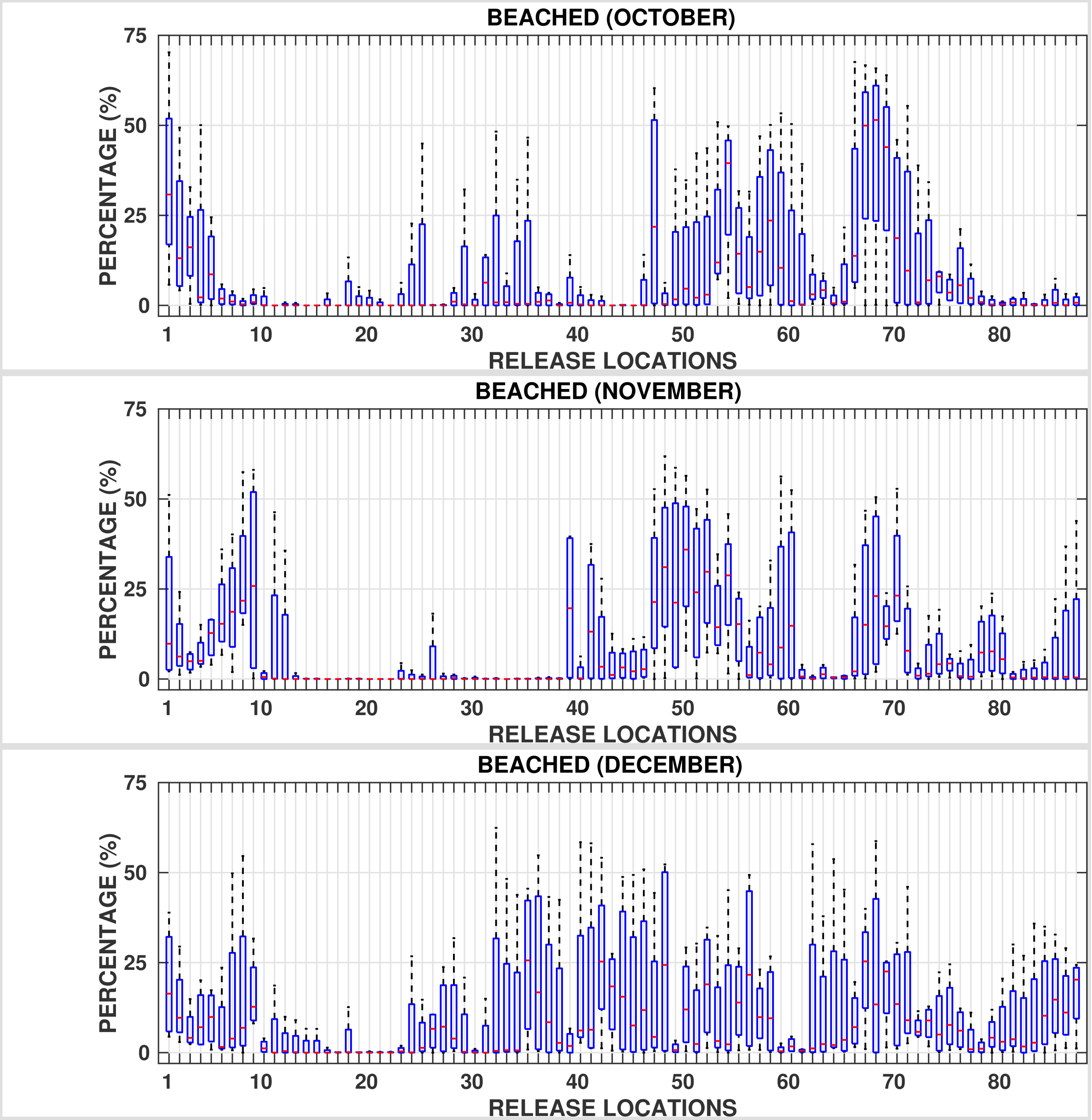

Supplementary Figure S4: Percentages of oil beached, twenty (20) days after the commencement of the spill for each release location ($RL_i \; : \; i = 1 : 87$) for all the four years considered in simulations.

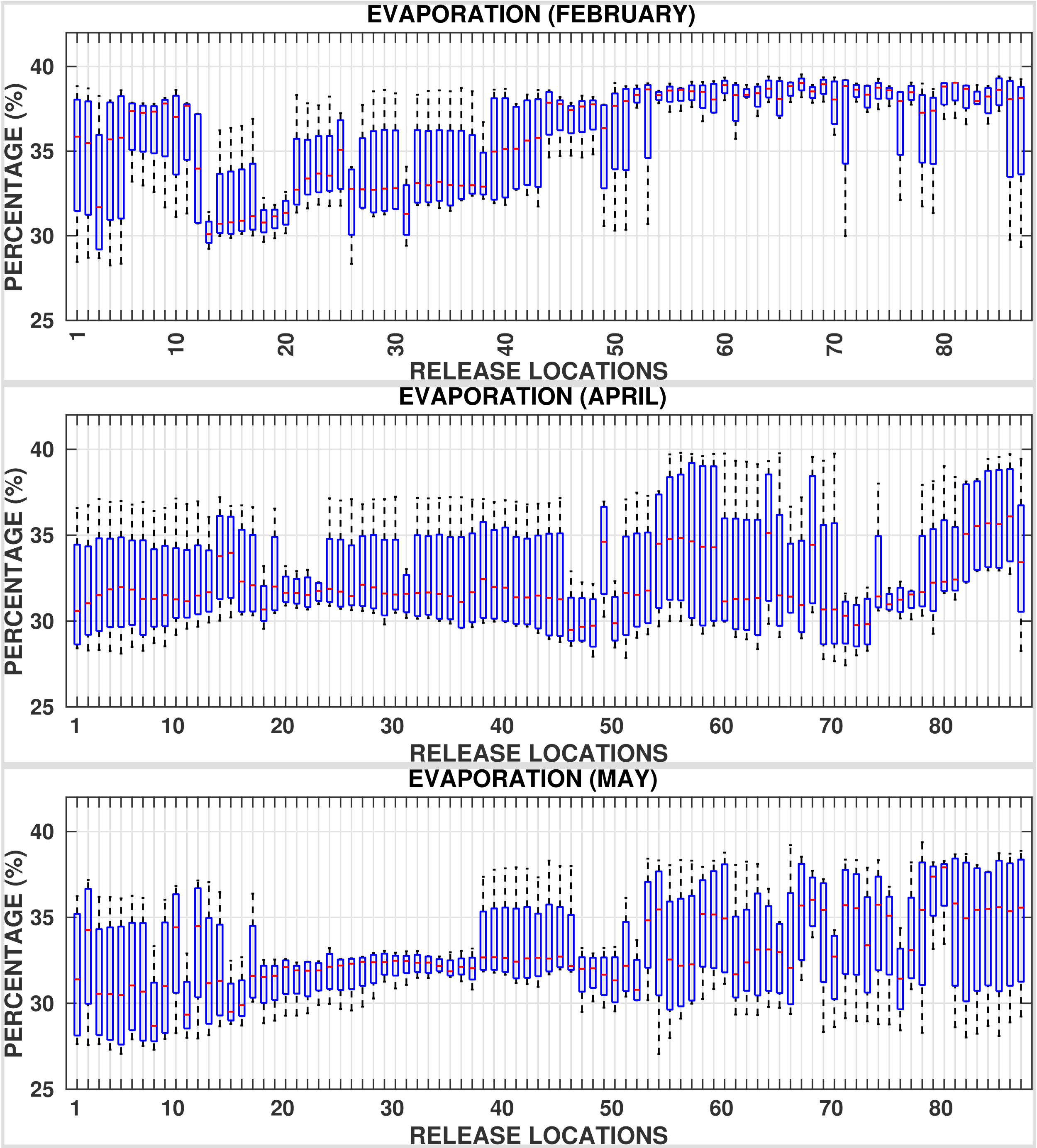

Supplementary Figure S5: Percentages of oil evaporated, twenty (20) days after the commencement of the spill for each release location ($RL_i \ : \ i = 1 : 87$) for all the four years considered in simulations.

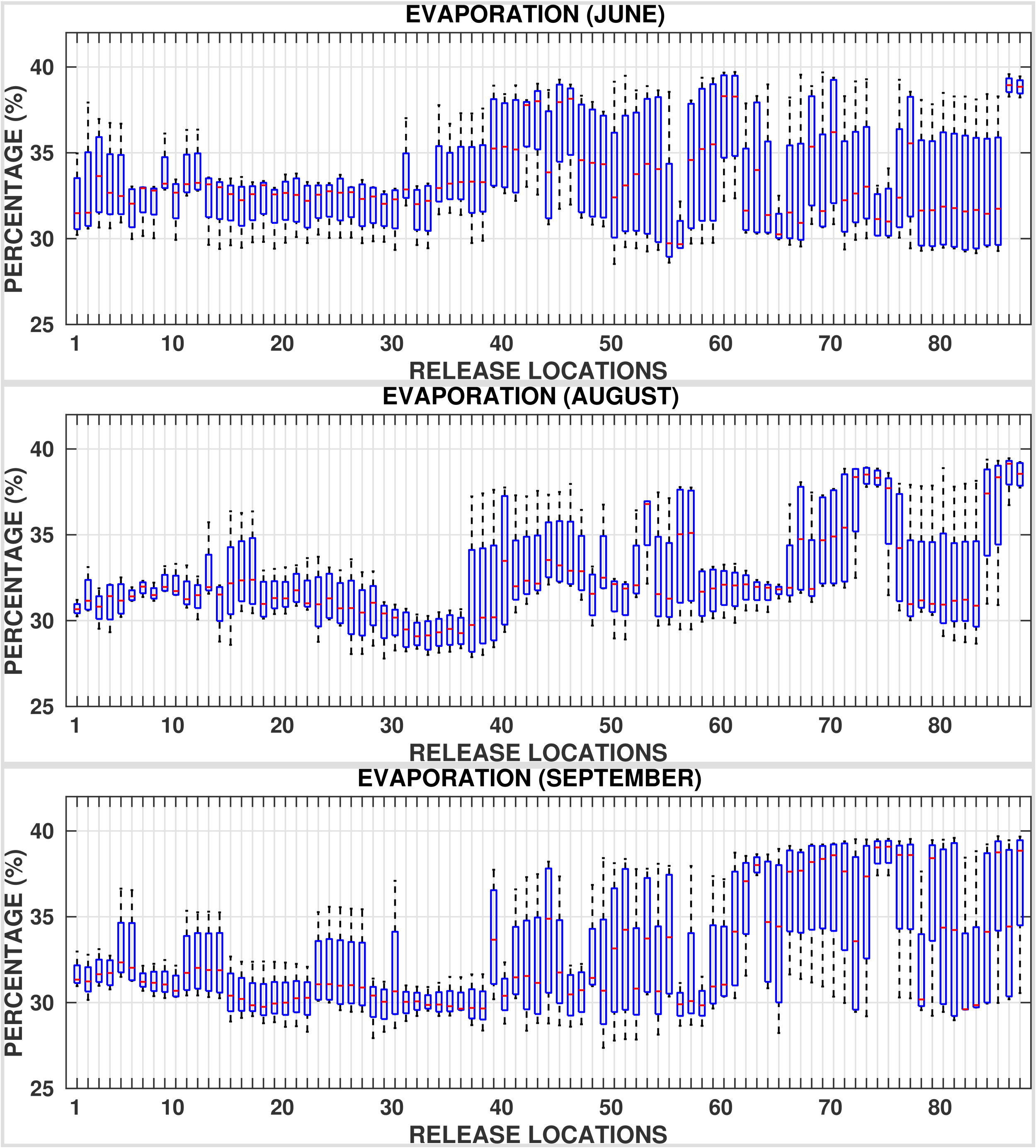

Supplementary Figure S5: Percentages of oil evaporated, twenty (20) days after the commencement of the spill for each release location ($RL_i \; : \; i = 1 : 87$) for all the four years considered in simulations.

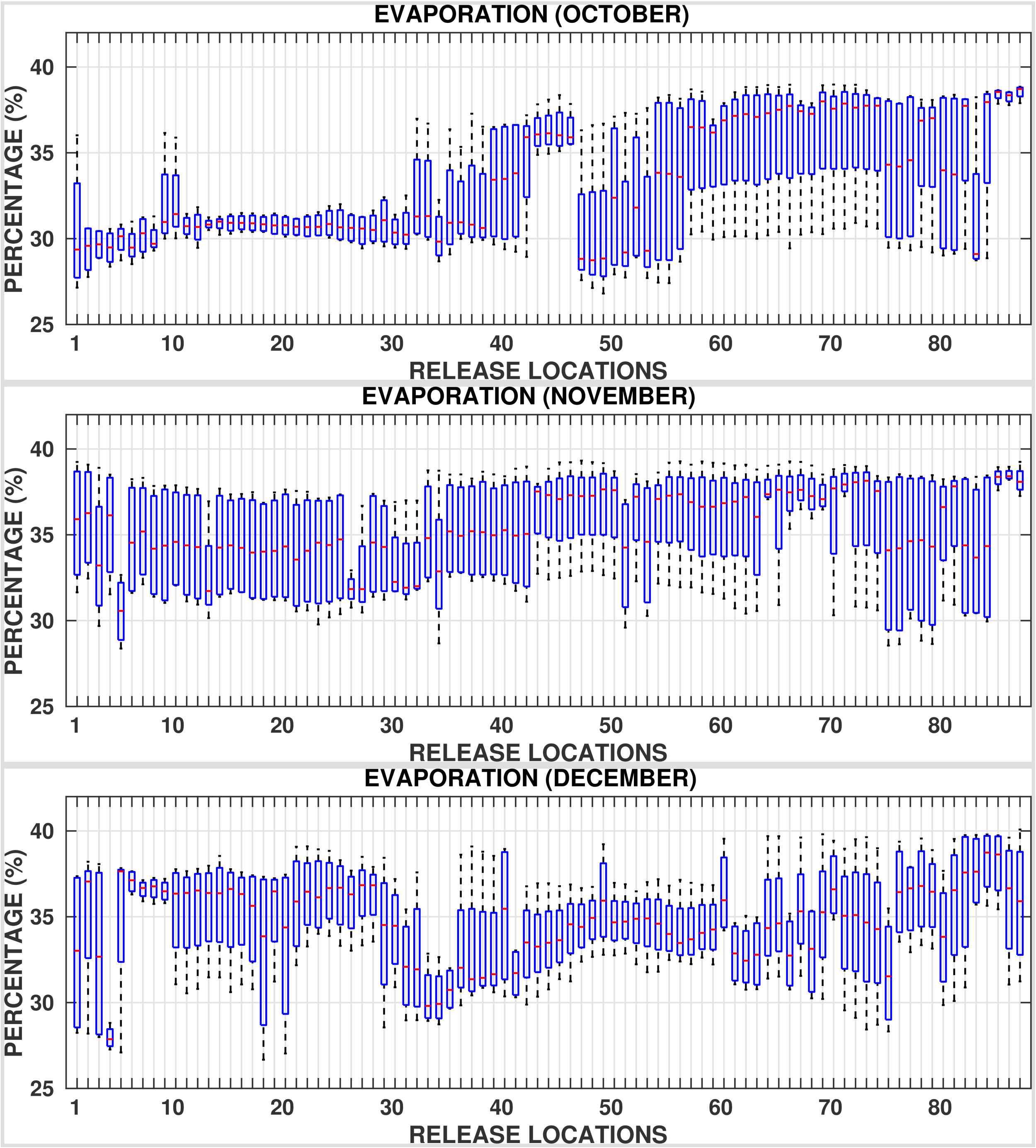

Supplementary Figure S5: Percentages of oil evaporated, twenty (20) days after the commencement of the spill for each release location ($RL_i \; : \; i = 1 : 87$) for all the four years considered in simulations.

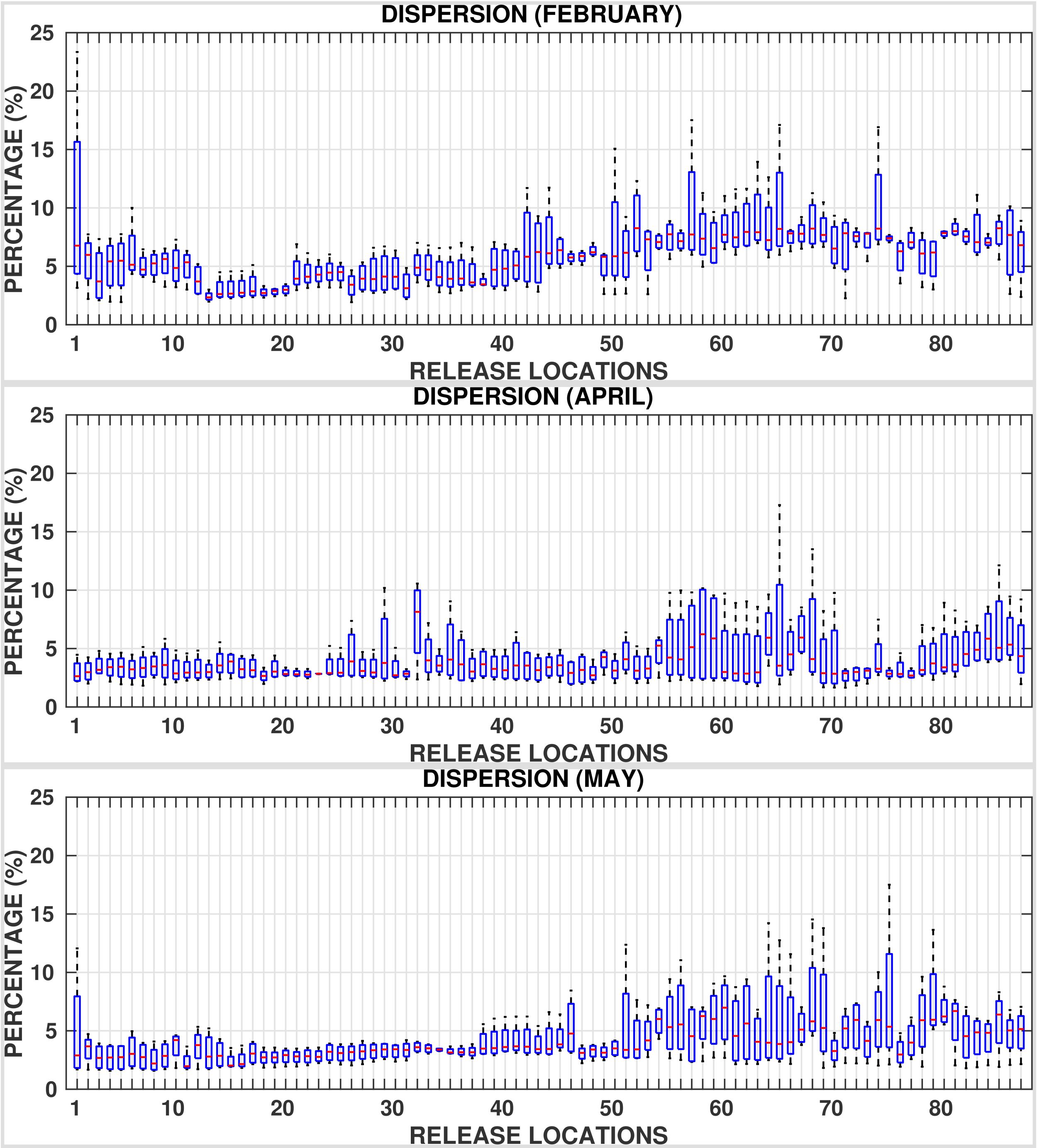

Supplementary Figure S6: Percentages of oil dispersed, twenty (20) days after the commencement of the spill for each release location ($RL_i \; : \; i = 1 : 87$) for all the four years considered in simulations.

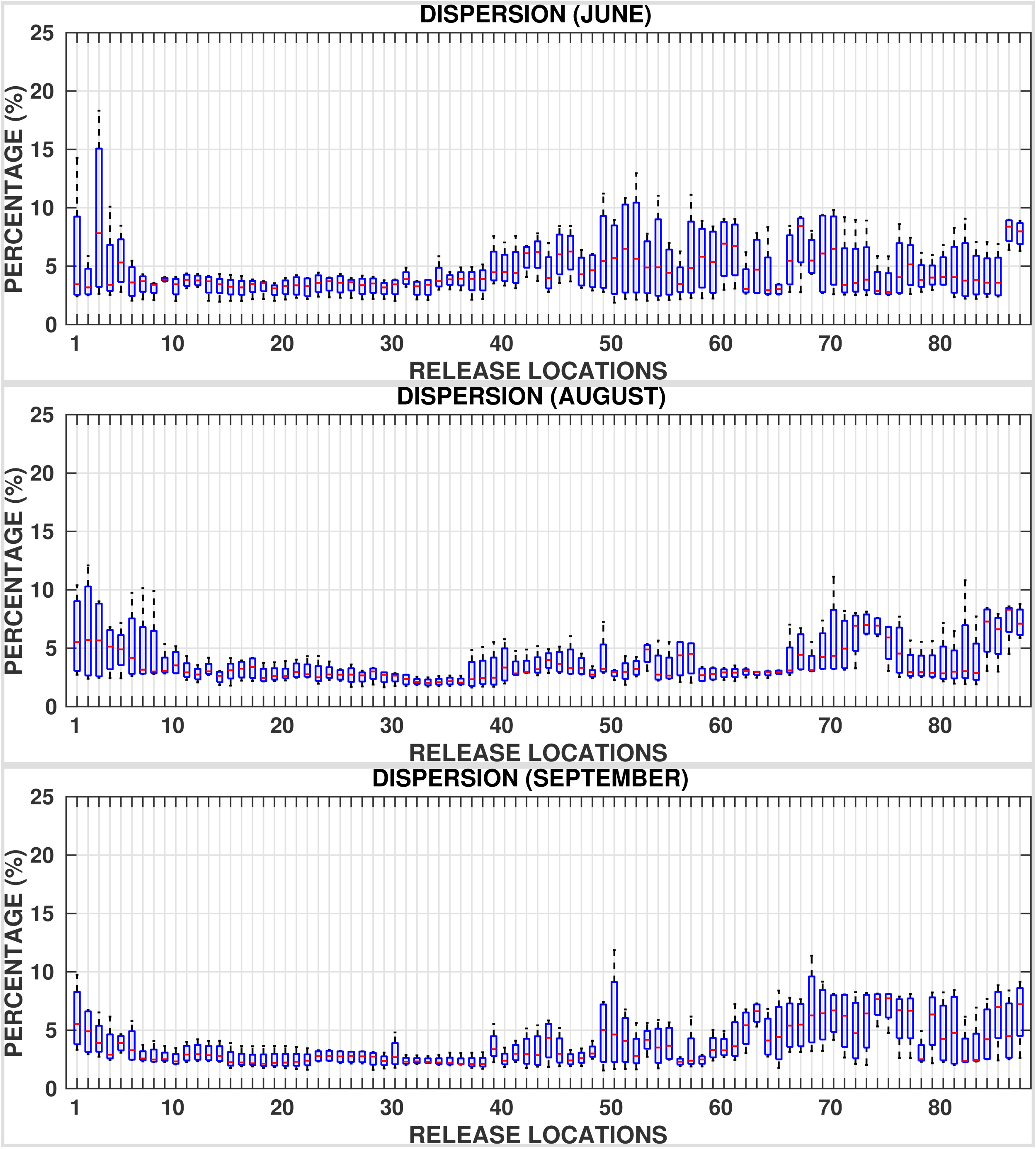

Supplementary Figure S6: Percentages of oil dispersed, twenty (20) days after the commencement of the spill for each release location (RL_i : $i = 1 : 87$) for all the four years considered in simulations.

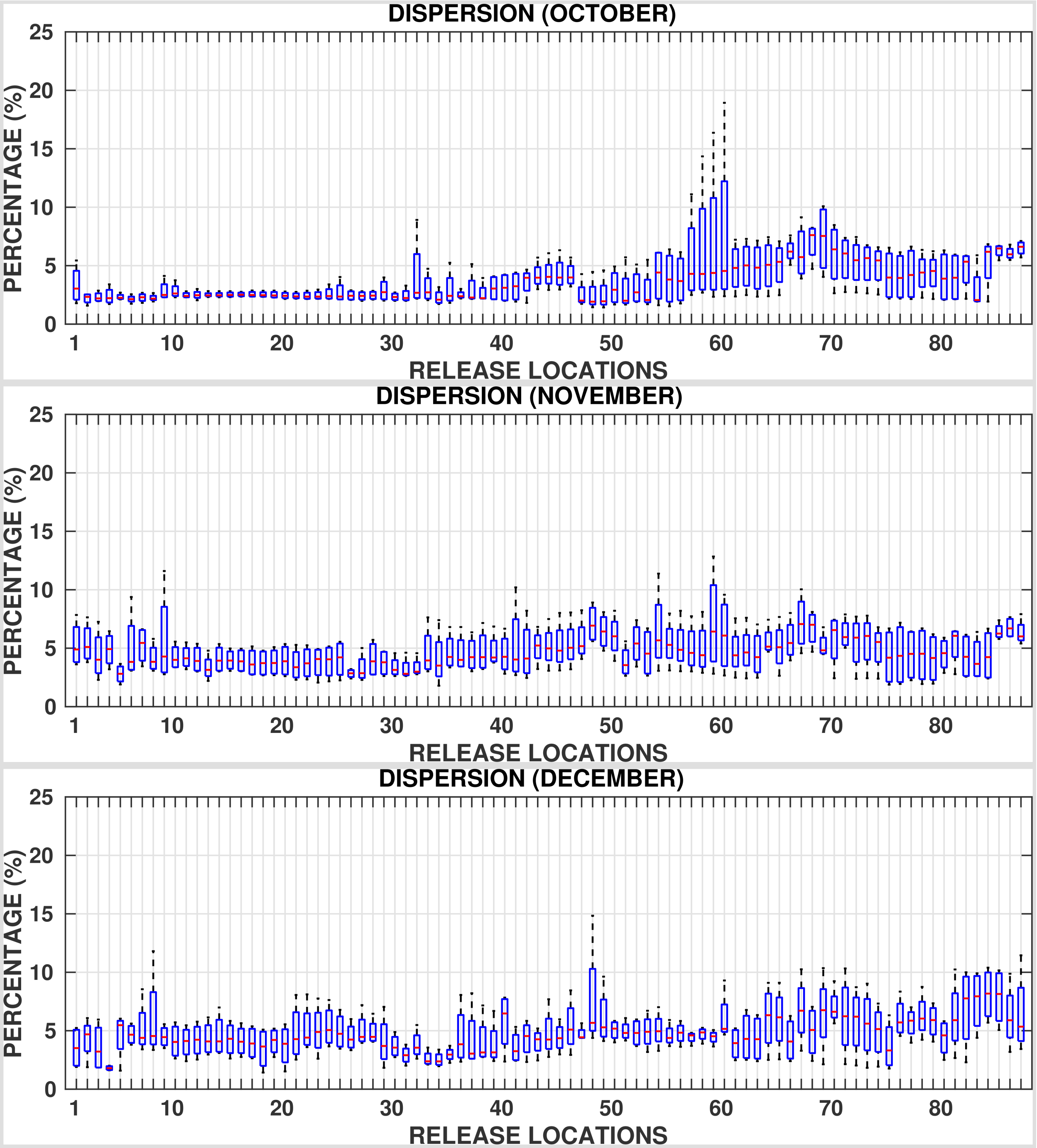

Supplementary Figure S6: Percentages of oil dispersed, twenty (20) days after the commencement of the spill for each release location ($RL_i \ : \ i = 1 : 87$) for all the four years considered in simulations.
